# Characterization of Organic Nitrogen by Chlorination, Ozonation, and Stable Isotope Analysis of Nitrate

**DOI:** 10.1021/acs.est.5c01034

**Published:** 2025-06-24

**Authors:** Jiwoon Ra, Kun Huang, Joachim Mohn, Thomas B. Hofstetter, Elisabeth Muck, Urs von Gunten

**Affiliations:** † Swiss Federal Institute of Aquatic Science and Technology, 28499 Eawag, Dübendorf, CH-8600, Switzerland; ‡ 28501 Empa, Swiss Federal Laboratories for Materials Science and Technology, Dübendorf CH-8600, Switzerland; § Institute of Biogeochemistry and Pollutant Dynamics (IBP), ETH Zürich, Zürich 8092, Switzerland; ∥ School of Architecture, Civil, and Environmental Engineering, École Polytechnique Fédérale de Lausanne, Lausanne 1015, Switzerland

**Keywords:** dissolved organic matter, amines, ozonation, chlorination, nitrate, nitrogen isotopes

## Abstract

During oxidation, nitrogenous species in dissolved organic matter (DOM) are critical in the formation of nitrogenous, potentially toxic disinfection byproducts, but their chemical identity remains poorly understood. Here, we developed three complementary approaches to identify and quantify reactive amines in model compounds and DOM, including aliphatic primary and secondary amines, aryl-type primary amines, amino acids, and terminal peptidic amino groups. With the chloramine formation assay, the total reactive amines were quantified for the main subgroups. An assay with continuous ozonation quantified three types of reactive amines based on nitrate formation rate constants (*k*_NO_3_‑_): *k*_NO_3_‑_ < 0.1 M^–1^ s^–1^ for secondary and aliphatic primary amines; *k*_NO_3_‑_ = 0.9–1.9 M^–1^ s^–1^ for aryl-type primary amines; *k*_NO_3_‑_ = 15–110 M^–1^ s^–1^ for amino acids and peptidic amino groups. The analysis of ^15^N/^14^N ratios of nitrate helped to distinguish reactive amines based on ^15^N enrichment factors (ε_N_): aliphatic (or aryl-type) primary amines (ε_N_:-9 to -3‰), and amino acids and peptidic amino groups (ε_N_:-28 to -19‰). Overall, 23–27% of the organic nitrogen in DOM isolates comprises oxidant-reactive amines, with 5–6% secondary amines, 10–14% aliphatic primary amines, 4% aryl-type primary amines, 1–4% amino acids, and 0–2% peptidic amino groups. Based on the quantitative characterization of amine moieties in DOM, which are possible precursors of N-DBPs, the formation potential of N-DBPs upon oxidative water treatment was estimated.

## Graphical abstract

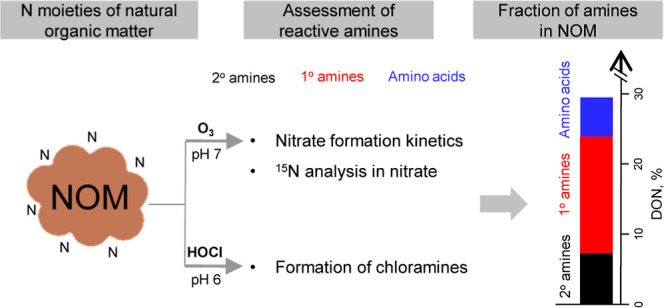

## 1 Introduction

Dissolved organic matter (DOM) is a complex mixture of cross-linked organic compounds and is ubiquitous in source water for drinking water supply and municipal/industrial wastewater effluents.^1,2^ During oxidative water treatment, chemical oxidants (e.g., chlorine, ozone or chlorine dioxide) react with electron-rich DOM moieties such as phenols and amines, thereby reducing oxidation/disinfection efficiency.^3^ As a consequence of the reactions between DOM and chemical oxidants, potentially toxic disinfection byproducts (DBPs) are formed such as trihalomethanes (THMs), haloacetic acids (HAAs), halonitromethanes (HNMs), haloacetonitriles (HANs), *N*-nitrosamines, etc.^4–9^ While carbonous DBPs (C-DBPs; THMs and HAAs) are regulated, nitrogenous DBPs (*N*-DBPs; HNMs, HAN, and *N*-nitrosamines) are not, despite their higher toxicity.^10–13^ *N*-DBPs formation is expected to increase in the context of water reuse, in conjunction with treatment of wastewater-impaired and eutrophic source waters because of high concentrations of dissolved organic nitrogen (DON).^3,14,15^ The concentrations of DON span from 0.2 to 0.3 mgN/L for source water to 0.6–1.4 mgN/L for municipal wastewater effluents.^16–18^ A fraction of large biopolymers and humic substances constitutes a major pool of DON, while hydrophilic base moieties are primary precursors of *N*-DBPs, indicating a distinct chemical nature between these fractions.^18–20^ However, the chemical identity of DON moieties is still poorly understood due to a lack of methods for their identification and quantification.

A detailed structural characterization of functional moieties within DON is currently impossible. In source waters, up to 20% of nitrogen composition in DON is estimated to originate from amino acids and peptidic amino groups (*N* in primary amines).^15,21^ The N-fraction of reactive primary and secondary amines seems in the range of 20–40% of DON,^22^ but other constituents such as (poly)­peptides (*N* in amides), nitrogen-containing heterocycles, and nucleotides also contribute to DON.^15,21^ In wastewater effluents, a similar fraction (<20%) of DON is composed of amino acids and peptidic amino groups, while the sum of synthetic organic chemicals (e.g., ethylenediaminetetraacetic acid, dimethylamine, nitrilotriacetic acid, caffeine, etc.) contributes <5%.^14,23^ So far, only a small fraction of the nitrogen-containing moieties in DOM have been identified, resulting in a significant lack of understanding of its chemical composition.

Nitrogenous moieties in DOM and their reaction products from chlorination and ozonation have been studied previously.^22,24,25^ Possible reactive nitrogenous structures include 1°/2°/3° amines, imines, amides, nitriles, heterocycles in aliphatic compounds, or polymeric structures. Among these nitrogenous functional groups, amines are recognized as major precursors of *N*-DBPs in reactions with chemical oxidants.^6,26–28^ Amines react with chlorine with apparent second-order rate constants in the range of 10^2^–10^4^ M^–1^ s^–1^ at pH 7 to form chloramines.^29–31^ Chloramines can be detected by colorimetric methods,^32^ wherefore, total chloramines enable a quantification of the chlorine-reactive amine concentrations in DOM. Ozone reacts with amines at pH 7 with apparent second-order rate constants in the range of 10^1^–10^4^ M^–1^ s^–1^,^30^ to, e.g., *N*-oxides, nitroalkanes, and oximes, which can be transformed to nitrate as the end product of ozonation.^22,24,25,33^ Nitrate formation rates depend on the types of amine moieties (i.e., amino acids and aliphatic primary amines), allowing us to distinguish them in DOM for different ozone exposures.^22^ A combined approach utilizing chlorination and ozonation was previously applied to estimate the concentrations of amine moieties in DOM, based on formations of chloramines and nitrate.^22^ However, it is unclear which organic amine moieties (1°/2°/3° amines, amino acids, peptidic amino groups, etc.) are present in DOM and what their fractions are. Furthermore, selective tools are needed to specifically identify different types of amine moieties because nitrate is the end product of not only amines but also partially oxidized nitrogenous compounds (e.g., nitroalkanes and oximes) during ozonation.^22^

Amine moieties in DOM can also be evaluated by stable isotope analysis (SIA). Small changes of the isotopic compositions in oxidation products are often indicative of differences in precursors or formation mechanisms.^34–37^ For example, for *N*-nitrosodimethylamine (NDMA) formed from ranitidine during chloramination, trends of ^15^N/^14^N, ^13^C/^12^C, and ^2^H/^1^H ratios reflected the reaction sequences and isotope effects of bond cleavage and formation processes, leading to a better mechanistic understanding.^38–40^ Similar approaches were used to disentangle the formation of hydrogen peroxide during ozonation of phenols and olefins and the formation of chloroform during chlorination of phenolic compounds and α,β-diketones.^41,42^ Therefore, we hypothesize that the *N*-isotopic composition of nitrate from ozonation of nitrogenous moieties in DON might provide insights into reaction pathways given that procedures for SIA of nitrate from aqueous matrices are established.^43,44^

The aim of this study was to characterize oxidant-reactive amine moieties in DOM. Different types of reactive amine moieties, including aliphatic primary and secondary amines, aryl-type primary amines, amino acids, and peptidic amino groups, were quantified using both a chloramine formation assay and a nitrate formation kinetics assay during continuous ozonation. In the chloramine formation assay, both mono- and dichloramines and their stability were evaluated. In the nitrate formation kinetics assay, rate constants specific to each type of amine were determined. These findings were combined with SIA of nitrate from ozonation, yielding ^15^N enrichment factors specific to the amine precursor. Based on a quantitative characterization of amine moieties in DOM obtained in this study, the formation potential of *N*-DBPs upon oxidative water treatment was estimated.

## 2 Materials and Methods

### 2.1 Standards and Reagents

All chemicals and reagents were of the highest available purity, prepared with ultrapurified water (≥18.2 ΩM/cm, Millipore Milli-Q water purification system), and are listed in Table S1 (Supporting Information). Twenty-two model *N*-compounds were selected (Text S1) based on their substituents (e.g., ethyl, benzyl, or no carbon chains) or other substituents (e.g., carboxylic acid and halogen atoms). These compounds include two aliphatic or aryl-type secondary amines, nine amino acids, two polypeptides, four aliphatic primary amines, two aryl-type primary amines, two nitroalkanes, and one oxime. Four natural organic matter (NOM) isolates were used in this study, including Suwannee river NOM (SRNOM), Upper Mississippi river NOM (UMRNOM), Suwannee river fulvic acid (SRFA), and Nordic lake fulvic acid (NLFA), all from the International Humic Substances Society.^45^ Chlorine stock solutions were prepared from a sodium hypochlorite solution (4–5% available chlorine, Sigma-Aldrich) and the concentrations were determined spectrophotometrically at 292 nm (ε = 350 M^–1^ cm^–1^) at pH 11 prior to use.^46^

### 2.2 Chloramine Formation Assay

To measure chloramine forming-amine moieties, diethyl-*p*-phenylenediamine (DPD) with potassium iodide (KI) was used in the presence of free available chlorine (FAC). FAC was dosed in approximately 10× molar excess (final concentration 80 μM) to the solution containing model *N*-compounds (or NOM isolates) to convert reactive amine moieties to chloramines (Figure S1a) (5 mM phosphate, pH 6). The concentrations of residual FAC, monochloramine, and dichloramine were sequentially determined by spectrophotometric analysis at 510 nm by injection of 2 mM DPD with varying final concentrations of KI: 0 μM, 60 μM, and 40 mM, respectively (details in Text S2 and Figure S2).

### 2.3 Nitrate Formation Kinetics Assay

Continuous ozonation of model *N*-compounds or NOM isolates was conducted at pH 7 (10 mM phosphate) by sparging the reaction solution with ozone-containing oxygen to fully convert organic nitrogen to nitrate (Figures S1b, S3, S6 and Text S3). Hydroxyl radicals were scavenged using 50 mM tertiary butyl alcohol (*t*-BuOH). Samples were collected at specific intervals, quenched with excess 3-buten-2-ol, and analyzed for nitrate via HPLC (Ultimate 3000, Dionex).

### 2.4 Stable Isotope Analysis

To measure ^15^N/^14^N isotope ratios of nitrate, aqueous nitrate samples were converted to gaseous nitrous oxide (N_2_O) first, which was analyzed either by an isotope ratio mass spectrometer (IRMS) or infrared laser spectroscopy.^43,47,48^ Stable isotope ratios are typically described with the delta (δ) notation (e.g., δ^15^N), which quantifies the relative abundance compared to a reference isotope standard (details in Text S4). This study applied two methodologies, detailed in Text S5. Comparison of both techniques resulted in comparable results, within the uncertainties. We therefore concluded that a potential enhancement in δ^17^O–NO_3_^–^, inherited from ozone, did not significantly affect δ^15^N– NO_3_^–^ analysis by IRMS.

### 2.5 Analysis of Initial δ^15^N of Analytical Standards

δ^15^N values of model *N*-compounds and NOM isolates were analyzed by an elemental analyzer-isotope ratio mass spectrometer (Table S3, δ^15^N: -60.9 to +13.8‰).

### 2.6 Analysis of δ^15^N in Nitrate Produced from the Ozonation of NOM Isolates

Due to the low nitrate concentrations from NOM ozonation (e.g., <0.2 μM/mgC/L for SRNOM), ^15^N/^14^N isotope ratios were determined by batch conversion-cavity ring-down spectroscopy (CRDS), which has a lower quantification limit (1 μM as nitrate, Text S6 and Figures S7–S10). To secure sufficient isotopic data (1–10 μM nitrate), ozonation was conducted with high NOM concentrations (∼50 mgC/L). To minimize matrix effects on [N_2_O] and δ^15^N (Figure S11), powdered activated carbon was used for NOM removal (Figure S12 and Text S7).

### 2.7 Determination of the Nitrogen Isotope Enrichment Factor (ε_N_) from δ^15^N

Isotope enrichment factor (ε) represents the trend of isotope fractionation in a certain type of reaction. In this study, the ^15^N/^14^N ratio of nitrate is indicative of nitrogen isotope fractionation in the transformation processes occurring from amine to nitrate. The ε_N_ could be obtained by fitting eq 1 (using Sigma Plot) derived from a previous study^37^

1 $$\left(\delta^{15}N_{NO3-}+1\right)/\left(\delta^{15}N_{precursor}+1\right)=\left(1-f^{\left(\varepsilon N+1\right)}\right)/\left(1-f\right)$$

where δ^15^ *N*_precursor_ and δ^15^ *N*_NO3‑_ denote the δ^15^ *N* values of the precursor and nitrate, and *f* = [precursor]_*t*_/[precursor]_0_.^37^ *f* was calculated from the molar nitrate concentration measured by HPLC-UV, assuming nitrate formation as the sole N sink ([precursor]_*t*_ + [NO_3_^–^] = [precursor]_0_). Initial precursor concentrations are target values for model N-compounds, while final nitrate concentrations apply to NOM isolates.

## 3 Results and Discussion

### 3.1 Application of the Oxidative Assays and SIA to Characterize Model *N*-Compounds

Three complementary assays (chloramine formation assay, nitrate formation kinetics assay, and SIA of nitrate) were first validated by using various individual and mixed model *N*-compounds to distinguish different reactive amine moieties.

#### 3.1.1 Chloramine Formation Assay

Figure 1 presents the results of the chloramine formation assay for two model *N*-compound mixtures and two NOMs (SRNOM, SRFA) with or without diethylamine, gly–gly, and glycine. Results for individual model *N*-compounds are provided in the Supporting Information (Table S4).

**Figure 1:**
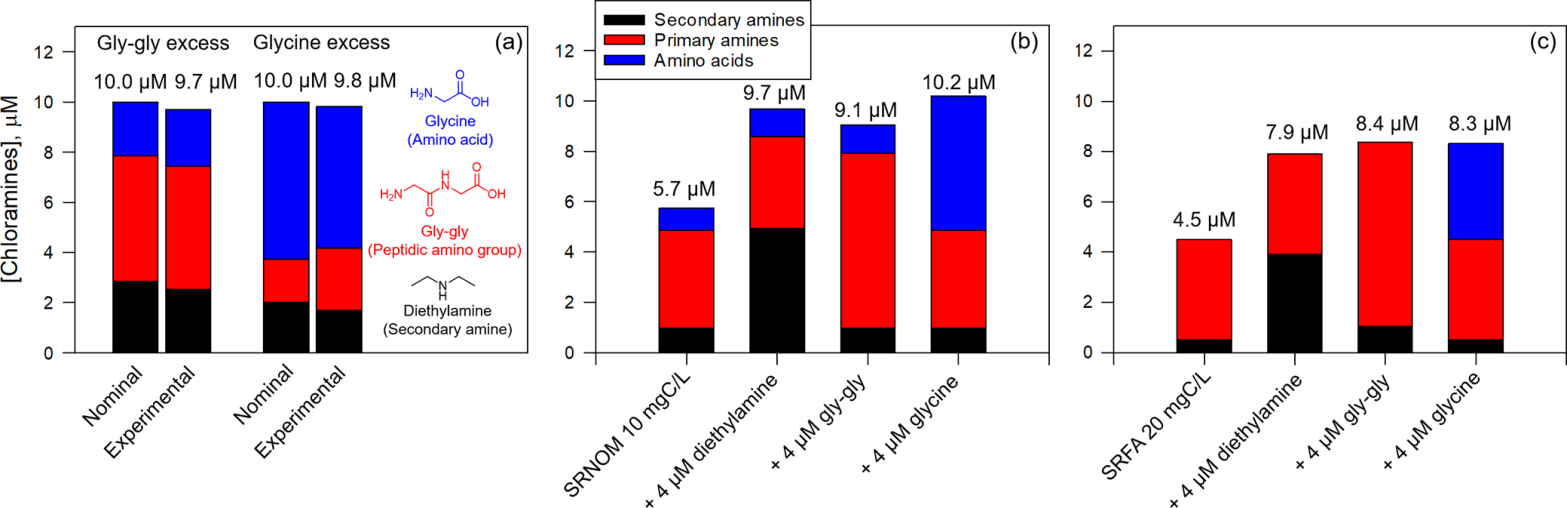
Screening of amine moieties in model *N*-compounds and NOM with the chloramine assay. (a) Mixtures of model *N*-compounds, (b) SRNOM, and (c) SRFA in the absence and presence of model *N*-compounds (secondary amine, primary amine, amino acid). The mixtures “gly–gly excess” and “glycine excess” consist of 2.2, 5, 2.8 μM and 6.3, 1.7, 2 μM of glycine, gly–gly, diethylamine, respectively. The numbers above the bars (Figure 1a) represent the total chloramine concentrations, both theoretical (nominal) concentrations and experimental measurements obtained through the chloramine assay. Experimental conditions: [FAC] = 80 μM for 2 and 30 min, followed by [resorcinol] = 10 μM for 1 min, followed by [DPD] = 2 mM with [KI] = 0, 60 μM and 40 mM in sequence to measure [FAC], [*N*–Cl amine], and [*N*–Cl_2_ amine], respectively, at pH 6 (10 mM phosphate).

The total concentrations of amine moieties, along with individual types (secondary and primary amines and amino acids), showed good agreement between the nominal and measured concentrations of the selected model *N*-compounds (Figure 1a and Table S4). Note that peptidic amino groups behaved like aliphatic or aryl-type primary amines in the assay due to their stable chloramine forms, unlike amino acids.^49^ Amine moieties in mixtures of model *N*-compounds and NOM isolates (Figure 1b,c) demonstrated that the developed method effectively quantifies chloramine-forming amines with 101 ± 4% yields, even with NOM (Figure S13).

#### 3.1.2 Nitrate Formation Kinetics Assay

Nitrate formation rates of model *N*-compounds were assessed with continuous ozonation. The complex oxidation of model *N*-compounds to nitrate was simplified into an overall reaction (eq 2). The second-order nitrate formation rate constant (*k*_NO_3_‑_) was derived from ln­(1-[NO_3_^–^]/[model-*N*]_0_) vs ozone exposure (Ms) (eq 3).^22^

2 $$\text{model‐}N+O_{3}\to {NO}_{3}^{-}$$

3 $$\ln\left({1-[NO}_{3}^{-}]/{\left[\text{model‐}N\right]}_{0}\right)=‐k_{NO3-}\int \left[O_{3}\right]dt$$

where *k*_NO_3_‑_ is an apparent second-order rate constant, assuming nitrate as an exclusive final *N*-product from model *N*-compound ozonation (Section 2.7).

Table 1 and Figure S14 show the apparent second-order rate constants, *k*_NO_3_‑_ of 20 selected *N*-compounds, which decrease in the order oxime (∼185 M^–1^ s^–1^) > peptidic amino group and amino acids (15–110 M^–1^ s^–1^) > γ-aminobutyric acid and gabapentin (7–8 M^–1^ s^–1^) > primary amines (0.9–1.9 M^–1^ s^–1^ for aryl-type, <0.1 M^–1^ s^–1^ for aliphatic) ≈ nitroalkane (1.5 M^–1^ s^–1^ for aryl-type, <0.1 M^–1^ s^–1^ for aliphatic). Two secondary amines (diethylamine and *N*-benzylmethylamine) were not tested for *k*_NO_3_‑_ because they usually have lower nitrate yields than their primary amine analogues.^22,50^

**Table 1:**
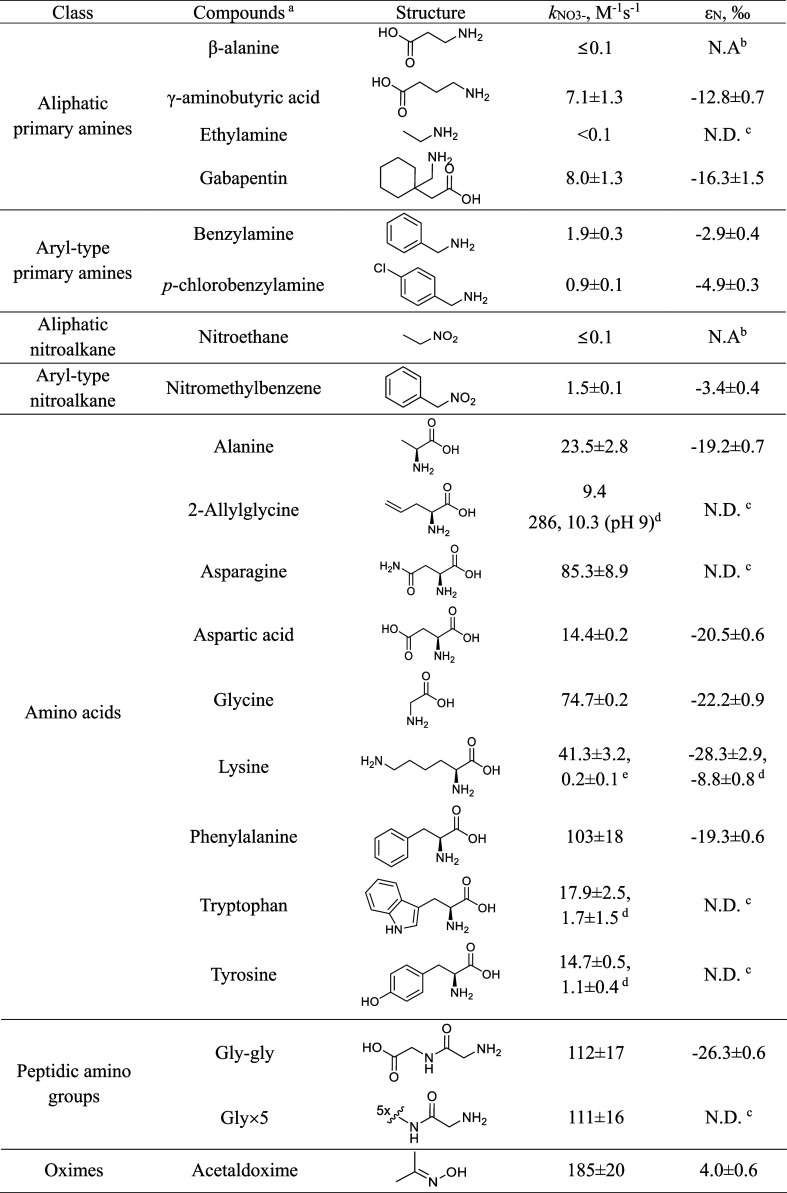
Summary of Apparent Second-Order Nitrate Formation Rate Constants (*k*_NO_3_‑_) and ^15^N Enrichment Factors (ε_N_) for Continuous Ozonation of Model *N*-Compounds at pH 7 (5 mM Phosphate) if Not Otherwise Indicated^f^

a Within compound classes, model *N*-compounds are in alphabetical order.

b Not available: no ε_N_ can be provided because reactions with hydroxyl radical yields other products than nitrate at high ozone exposures (∼50 Ms) (for more details see Section 3.1.3 and Figure S15).

c Not determined.

d Biphasic trend from intact amino acids, followed by amino acids structurally altered at the side chain (see Figure S14).

e Biphasic trend from amino acids, followed by aliphatic primary amines at the side chain (see Figure S14).

f The model *N*-compounds are grouped according to compound classes (e.g., aliphatic primary amines, amino acids, etc.). For measurement of *k*_NO3‑_ and ε_N_, fixed ([model-*N*] = 5 μM) or varying ([model-*N*] = 50–250 μM) initial concentrations of model *N*-compounds were used, respectively, in the presence of 50 mM *t*-BuOH (see Figures S14 and S15). The error ranges denote the ranges of experimental duplicates for *k*_NO3‑_ and the 95% confidence interval from fitting eq 1 for ε_N._

Previous studies classified nitrate formation yields from ozonation across functional groups (e.g., amino acids, aliphatic primary amines).^19,41^ However, the interpretation of the yields is compromised by its dependence on the reaction conditions (e.g., ozone exposure). In contrast, the nitrate formation rates obtained in this study during continuous ozonation serve as proxies for different N-containing functional groups in the model *N*-compounds and their mixtures.

Biphasic trends of nitrate formation were observed upon continuous ozonation of complex model *N*-compounds and mixtures used as surrogates for complex NOM isolates, such as lysine (Figure 2a) and a 1:1 molar mixture of phenylalanine and *p*-chlorobenzylamine (Figure 2b).

**Figure 2:**
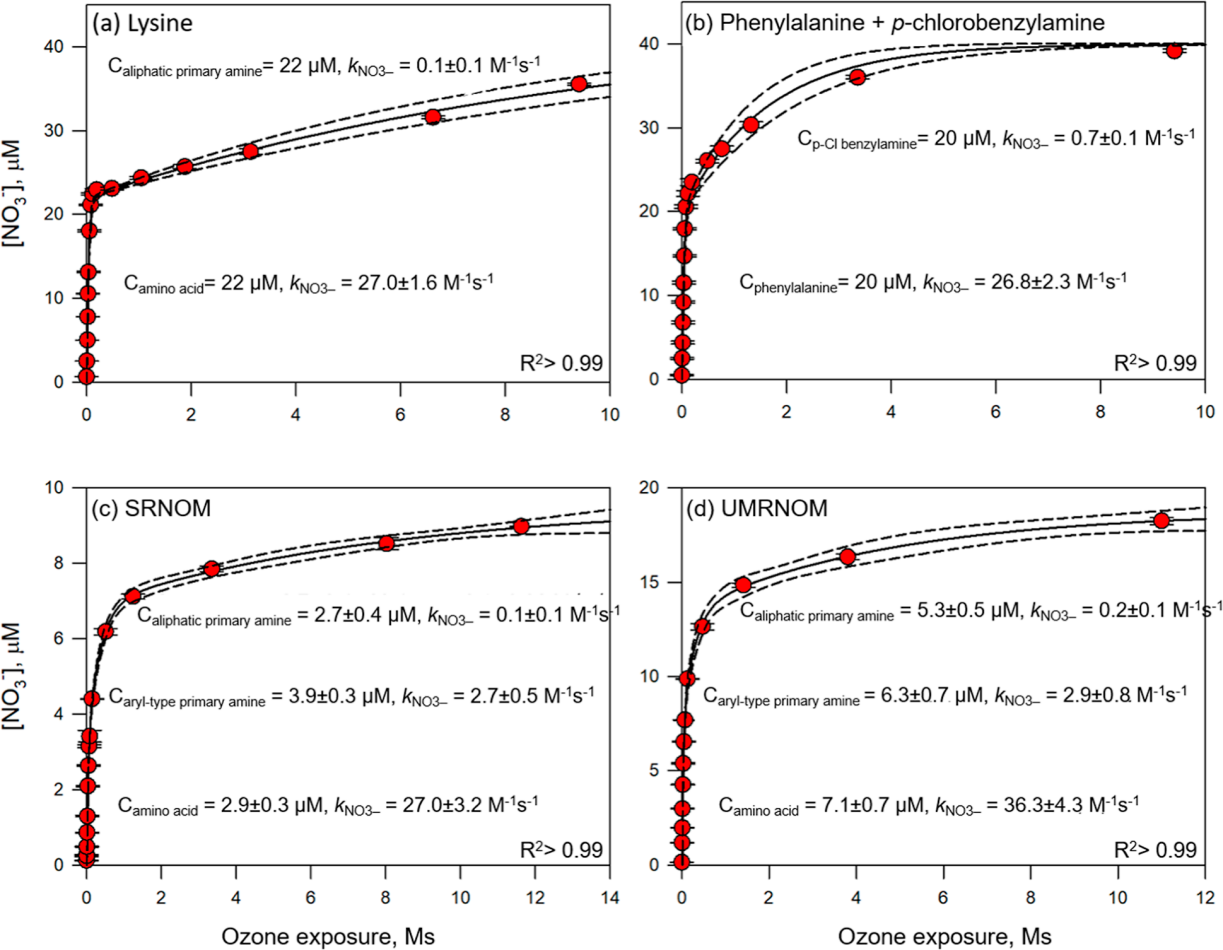
Continuous ozonation of (a) lysine, (b) mixture of phenylalanine and *p*-chlorobenzylamine, (c) SRNOM, and (d) UMRNOM. Evolution of nitrate as a function of the ozone exposure at pH 7 (10 mM phosphate). Error bars denote the standard deviations of analytical duplicates. Solid lines in (a–d) denote fitting models (Sigma Plot) of data points (see Text S3) corresponding to [NO_3_^–^] = *a*×(1-exp^–b*x*^)+*c*×(1-exp^–d*x*^) or [NO_3_^–^] = *a*×(1-exp^–b*x*^)+*c*×(1-exp^–d*x*^)+*e*×(1-exp^–f*x*^), respectively. X denotes the ozone exposure and (a–f) represent initial concentrations and *k*_NO_3_‑_ for the moieties of amino acids (or peptidic amino groups), aryl-type primary amines, and aliphatic primary amines in DOM, respectively. Dashed lines represent 95% confidence intervals. Experimental conditions: [Lysine] = 22 μM, [phenylalanine] = [*p*-chlorobenzylamine] = 20 μM, [SRNOM] = [UMRNOM] = 50 mgC/L, ozone generator power = 8% for 20 min, followed by an increase to up to 100% for 5 h, in the presence of 50 mM *t*-BuOH at pH 7, 5 mM phosphate buffer.

Plotting nitrate formation vs ozone exposure and fitting to eq 3 (Text S3) revealed distinct *k*_NO3‑_phases, with typical second-order rate constants for nitrate formation from amino acids (∼27 M^–1^ s^–1^), aliphatic primary amines (0.1 M^–1^ s^–1^, lysine), and aryl-type primary amines (0.7 M^–1^ s^–1^, *p*-chlorobenzylamine). This highlights how nitrate formation kinetics can characterize reactive amine moieties in complex *N*-compound mixtures during ozonation, an approach further evaluated for NOM isolates (Figure 2c,d).

#### 3.1.3 δ^15^N in Nitrate Formed from Model *N*-Compounds during Ozonation

To complement nitrate formation kinetics, the chemical nature of nitrogenous precursors was explored by ^15^N/^14^N ratio analyses of the formed nitrate. Trends in δ^15^N versus nitrate conversion yield were used to determine ε_N_ values for nitrate formation from individual model *N*-compounds. Table 1 and Figure S15 show a declining trend in ε_N_ values in the order oxime (4‰) > aryl-type primary amines (-3 to -5‰) ≈ aryl-type nitroalkane (-3‰) > γ-aminobutyric acid and gabapentin (-13 to -16‰) > peptidic amino groups and amino acids (-19 to -28‰) (Table 1). All model *N*-compounds exhibited normal isotopic fractionation for nitrate formation (i.e., ^15^N-depleted nitrate formed more rapidly), except acetaldoxime, which showed inverse fractionation (^15^N-enriched nitrate formed preferentially).^51,52^ Surprisingly, δ^15^N values of nitrate from nitroethane and β-alanine could not be fully interpreted by the model (eq 1), which assumes exclusive nitrate formation, because hydroxyl radicals also influenced the reaction (Figure S15a,e). These aliphatic *N*-compounds are converted to nitrate extremely slowly (∼10% over tens of hours) because their major intermediates, aliphatic nitroalkanes, exhibit low second-order rate constants for the reaction with ozone.^53^ This slow conversion potentially allows for side reactions with unknown isotopic effects (e.g., nitromethane formation) given a significant contribution of hydroxyl radical oxidation.

For complex model *N*-compounds, which contain more than one reactive amine moiety, biphasic trends of δ^15^N of nitrate were observed for lysine (Figure 3a) and a 1:1 molar mixture of phenylalanine and *p*-chlorobenzylamine (Figure 3b).

**Figure 3:**
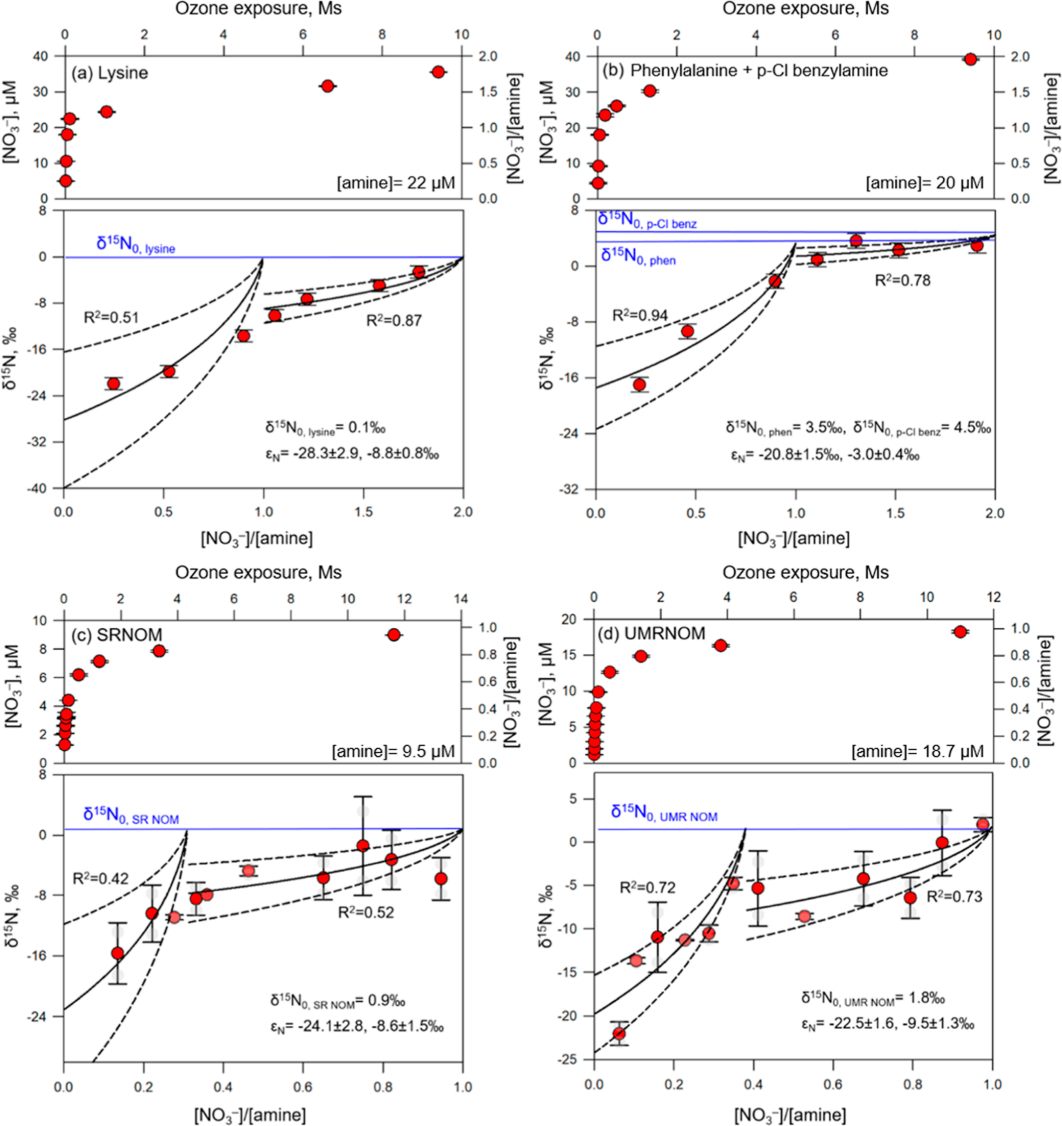
Continuous ozonation of (a) lysine, (b) mixture of phenylalanine and *p*-chlorobenzylamine, (c) SRNOM, and (d) UMRNOM. Evolution of nitrate (upper) and δ^15^N in nitrate (bottom) as a function of ozone exposure and amine conversion yield, respectively. Error bars denote the standard deviations for analytical duplicates. The solid lines represent ^15^N enrichment factors, and the dashed lines denote 95% confidence intervals of standard errors of ^15^N enrichment factors. For (a,b), amine concentrations at the onset of ozonation are known; for (c,d), amine concentrations were estimated as final nitrate concentrations derived from the model fit. Experimental conditions: [Lysine] = 22 μM, [phenylalanine] = [*p*-chlorobenzylamine] = 20 μM, [SRNOM] = [UMRNOM] = 50 mgC/L, ozone power = 8% for 20 min, followed by an increase to ≤100% for 5 h, in the presence of 50 mM *t*-BuOH at pH 7, 5 mM phosphate.

δ^15^N values of nitrate from lysine ozonation display two isotope fractionation regimes: the amino acid moiety reacts first (ε_N_ = -28.3‰), followed by the aliphatic primary amine moiety in the side chain (ε_N_ = -8.8‰) (Figure 3a). A similar trend appears in a mixture of two benzylamines with different nitrate formation rate constants. For phenylalanine and *p*-chlorobenzylamine, ε_N_ values are -20.8‰ and -3.0‰ (Figure 3b). This biphasic trend aligns with nitrate formation kinetics for complex model *N*-compounds and mixtures. For example, the high *k*_NO_3_‑_ observed in the initial phase of nitrate evolution (upper panels in Figure 3) might be attributed to amino acids, which also resulted in high δ^15^N values (lower panels in Figure 3). This trend continues up to the point at which the increase in nitrate concentration levels off (e.g., [NO_3_^–^]/[amine] = 0.3–0.4 for NOM isolates and approximately 1 for complex model *N*-compounds). Subsequently, a second phase with lower *k*_NO_3_‑_ values emerges, likely driven by aromatic and aliphatic primary amines, corresponding to lower δ^15^N values. Overall, the ε_N_ values of the screened model *N*-compounds provide additional constraints for different mechanisms, which can be applied to distinguish *N*-containing precursors. A combined approach of nitrate formation kinetics and stable isotope analysis can provide complementary information on nitrogen moieties in complex mixtures of *N*-compounds.

### 3.2 Mechanistic Investigation of Nitrate Formation during Ozonation of Model *N*-Compounds

Scheme 1 shows the proposed reaction pathways for the ozonation of model *N*-compounds to nitrate.^22,33,50^

**Scheme 1:**
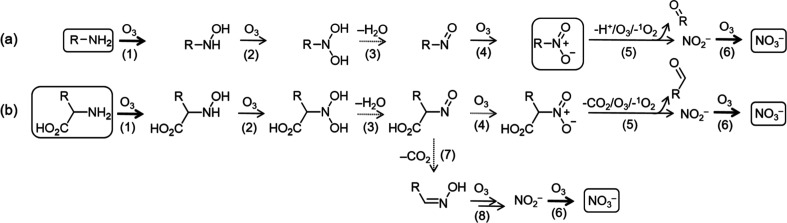
Proposed Reaction Pathways for the Ozonation of (a) Aliphatic (or Aryl-Type) Primary Amines and (b) Amino Acids;^22,33,50^ Framed Structures Stand for Reactants and Products that Were Quantified/Identified during Ozonation of Amines^a^

a Bold, plain, and dotted arrows denote confident, tentative, and speculative reaction steps, respectively.

For aliphatic or aryl-type primary amines, nitro products are formed as stable intermediates before nitrate is formed.^22,50^ Similar *k*_NO_3_‑_ values for benzylamine (1.9 M^–1^ s^–1^) and its nitro product, nitromethylbenzene (1.5 M^–1^ s^–1^), suggest a shared rate-limiting step in the ozone reaction (reaction 5, Scheme 1a). This can be further explained by the apparent rate constants at pH 7 for the reaction steps leading to nitrate (reactions 1–6, Scheme 1a). Here, the reaction of nitroalkane with ozone (reaction 5, Scheme 1a, *k* = 3.4 M^–1^ s^–1^) is significantly slower than the other reaction steps, thereby controlling the overall rate to nitrate formation.^22,50^ To explain the determined ε_N_, the reaction mechanism of the rate-limiting step should be considered. Because the nitro group is strongly electron withdrawing, the proton on the α-carbon becomes slightly acidic (p*K*_a_ for nitromethane = 10.2).^54^ It has been suggested that the deprotonated nitroalkane (i.e., nitroalkyl anion) reacts with ozone to an ozone adduct, leading to an aldehyde and nitrite.^33^ An ozone reaction on the α-carbon of the nitro functional group could explain the small value of ε_N_ for aryl-type primary amines (e.g., −2.9‰ for benzylamine) as nitrogen is not directly involved in the reaction (C–O bond formation), inducing a small nitrogen isotopic fractionation. This mechanistic hypothesis implies a secondary N isotope effect. Other reaction steps earlier in the reaction chain (reactions 1–4, Scheme 1a) are likely to have a minor effect on the δ^15^N of nitrate because most of the precursors are rapidly converted to nitroalkane before further transformation into nitrate occurs (reaction 5, Scheme 1a). The δ^15^N of nitroalkane might be considered identical to that of the precursor at the onset of nitrate formation, resulting in similar ε_N_ values for aryl-type primary amines and their corresponding nitroalkanes.

In contrast, amino acids and peptidic amino groups have *k*_NO_3_‑_ and ε_N_ of about 1 order of magnitude larger than those of aliphatic (or aryl-type) primary amines. Higher *k*_NO_3_‑_ for amino acids might result from a different ozone reaction mechanism, which involves an oxime intermediate with a significantly higher apparent second-order rate constant (*k*_O3_ ∼ 10^3^ M^–1^ s^–1^, reactions 7 and 8, Scheme 1b) than for nitro intermediates.^50^ However, the *k*_NO_3_‑_ of amino acids (e.g., 74.7 M^–1^ s^–1^ for glycine, Table 1) is still significantly lower than that for oximes, indicating another rate-limiting step with different ^15^N kinetic isotope effects determining the N isotope fractionation trends in nitrate. Given that the apparent second-order rate constants for reactions of amino acids (reaction 1, Scheme 1b, *k*_O_3__ ∼ 10^2^–10^3^ M^–1^ s^–1^)^30,55^ and *N*-hydroxyl amino acids with ozone (reaction 2, Scheme 1b, *k*_O_3__ ∼ 10^5^ M^–1^ s^–1^)^50^ at pH 7 are significantly higher than the *k*_NO_3_‑_ for amino acids, the most probable rate-limiting step is a dehydration of dihydroxylamine (reaction 3, Scheme 1b). During this reaction, nitrogen is directly involved (N–O bond cleavage), inducing a large nitrogen isotope fractionation (a primary N isotope effect).^56,57^ This mechanism leads to the large value of ε_N_ for amino acids (e.g., −22‰ for glycine) during ozonation at pH 7.

### 3.3 Application of the Developed Oxidative Assays and SIA to Characterize NOM Isolates

#### 3.3.1 Chloramine-Forming Amines in NOM Isolates

Chlorination was applied to classify the reactive amine compounds in four NOM isolates by the formation of mono- and dichloramines and considering their stabilities. Details on the procedure are provided in Text S2. Figure S17 shows the estimated concentrations of each reactive amine species in four NOM isolates as a function of the DOC concentrations (mgC/L). The measured concentrations of total chloramines exhibited an excellent correlation with DOC concentrations (*R* ^2^ ≥ 0.98). These concentrations are corrected values for residual ammonium (for details, see Text S2) already present in SRNOM and UMRNOM (Figure S18). The estimated concentrations of total chloramines for standard NOM isolates (0.49–0.77 μmolN/mgC) were higher compared to fulvic acids (0.25–0.30 μmolN/mgC). This observation agrees with the DON content (μmolN/mgC) of NOM isolates (≤3-fold higher for UMRNOM and SRNOM than for fulvic acids, Table S5). The trend in DON content for these NOM isolates is notably opposite to that of the phenol content, with fulvic acid exhibiting approximately twice the phenol content of standard NOM isolates.^58^ This highlights a distinct difference in the chemical composition between standard NOM isolates and fulvic acids, both of which are representative of natural organic matter.

For standard NOM isolates (Figure 4a,b) and fulvic acids (Figure 4c,d), the fractions of total chloramine-forming amines were in the range 23–32% of the total DON concentration. Even though the concentrations of chloramine-forming amines per DOC for NOM isolates varied by up to a factor of 3, the fraction of chloramine-forming amines normalized by the DON concentration remained relatively constant. The fractions of secondary and primary amines and amino acids for all four NOM isolates were 4–6%, 14–28%, and 0–4% of the total DON concentration, respectively. Apparently, the fraction of amino acids was negligible for both fulvic acids, indicating that the amino acid moiety likely originates from hydrophilic components, consistent with previous studies.^1,55,59^ Overall, it could be shown that primary amines are the major amine moieties in all NOM isolates, and the fractions of the primary amines (14–28%) and amino acids (0–4%) differ significantly for standard NOM isolates and fulvic acids.

**Figure 4:**
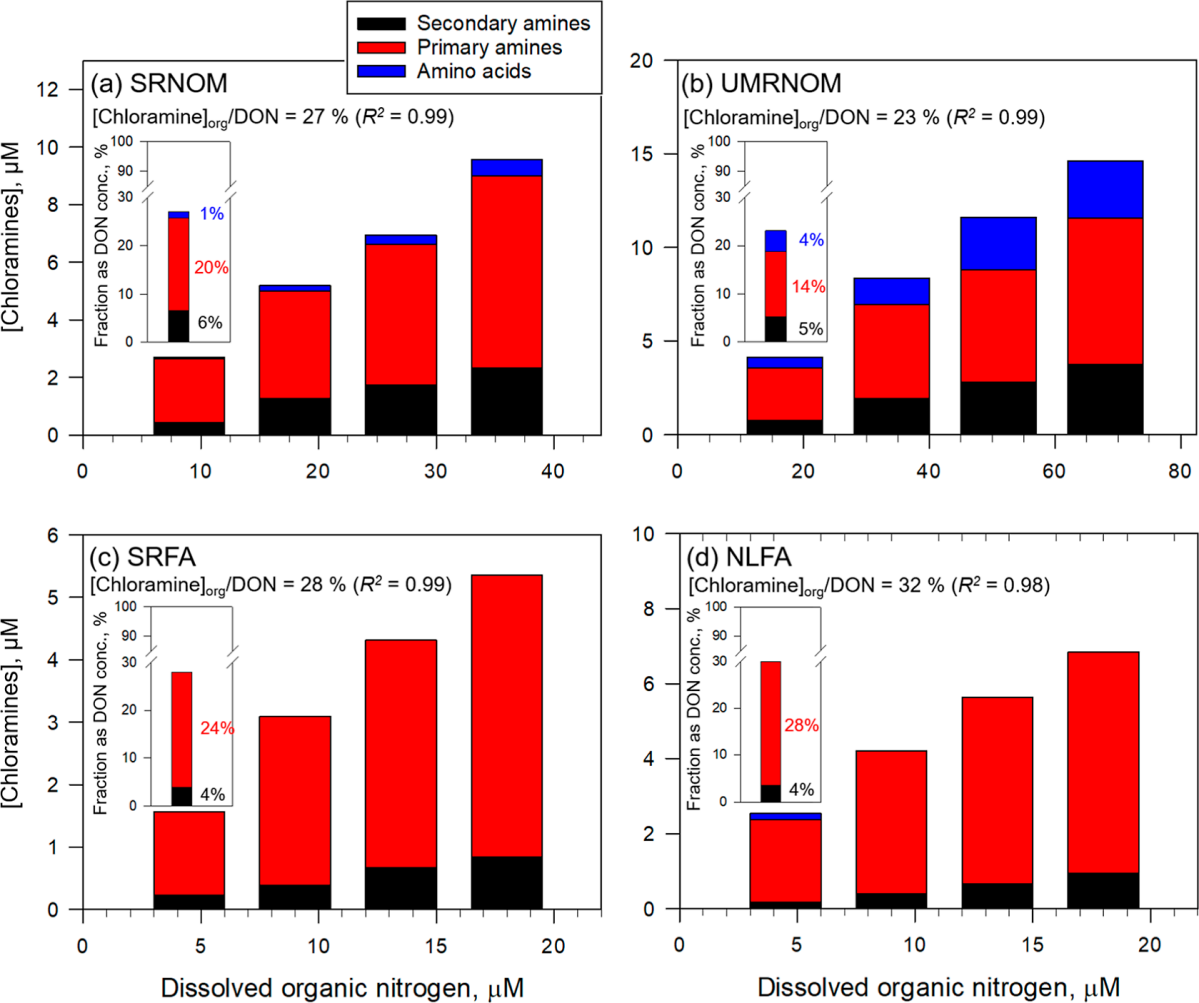
Chloramine formation assay: Screening of organic amine moieties in (a) SRNOM, (b) UMRNOM, (c) SRFA, and (d) NLFA as a function of the DON concentration (DOC concentration range 5–20 mgC/L, DON concentration was calculated based on the specific DON content, μmolN/mgC, Table S5)). The inset bar charts represent the average fraction of each organic amino moiety of the total DON concentration. Note that the interference of ammonium was corrected from the concentrations of amino acids and primary amines (see Text S2). Experimental conditions: [FAC] = 80 μM for 2 and 30 min, followed by addition of [resorcinol] = 10 μM for 1 min, followed by addition of [DPD] = 2 mM with [KI] = 0, 60 μM and 40 mM in sequence to measure [FAC], [*N*–Cl], and [*N*–Cl_2_], respectively, at pH 6 (10 mM phosphate).

#### 3.3.2 Nitrate-Forming Amines in NOM Isolates during Ozonation

Figure 2c,d shows the generation of nitrate as a function of the ozone exposure for SRNOM and UMRNOM in three distinct phases with different *k*_NO_3_‑_. For SRNOM (Figure 2c), organic nitrogen in amino acids or peptidic amino moieties were converted to nitrate first (2.9 μM and *k*_NO_3_‑_ = 27 M^–1^ s^–1^), followed by aryl-type primary amine moieties (3.9 μM and *k*_NO_3_‑_ = 2.7 M^–1^ s^–1^), and finally followed by aliphatic primary amine moieties (2.7 μM and *k*_NO_3_‑_ = 0.1 M^–1^ s^–1^). Note that the *k*_NO_3_‑_ value of 2.7 M^–1^ s^–1^ obtained from SRNOM is slightly higher than that of the aromatic primary amines in the model *N*-compounds. However, it can still be assigned to aromatic primary amine moieties, considering the range of *k*_NO_3_‑_ for different functional groups. For UMRNOM (Figure 2d), the composition of amine moieties and their *k*_NO3‑_ were similar to SRNOM, but the determined amine concentrations were higher due to its higher N content compared to SRNOM (Table S5).^45^ Figure S19a shows the formation kinetics of nitrate as a function of the ozone exposure during ozonation of the different NOM isolates. Nitrate formation is proportional to the DOC concentration (mgC/L, SRNOM) and the *N* composition in the NOM isolates (UMRNOM > SRNOM > NLFA ≈ SRFA). Figure S19b shows the nitrate concentrations normalized by the DON concentration of the NOM isolates. For a given NOM isolate, the normalized nitrate formation remains consistent across different NOM concentrations, suggesting that the nitrate formation rate constant might be independent of NOM concentration. The normalized nitrate formation is similar for all NOM isolates within a factor of 2 (3 to 6 μmol/mgN = 4–8% of DON). Nevertheless, amino acid (or peptidic amino) moieties, which almost completely convert to nitrate during the initial ozone exposure (e.g., 0.05 Ms), are more abundant in standard NOM isolates than in fulvic acids, which is in agreement with the results from chlorination.

Other fractions of the NOM isolate (e.g., amide moieties) apart from the amine moieties may interfere with nitrate formation. Therefore, nitrate formation from amide bonds in (poly)­peptides in NOM was tested in the presence/absence of a hydroxyl radical scavenger. Figure S20 shows that a primary amide is converted to nitrate during ozonation in the absence/presence of a hydroxyl radical scavenger. However, secondary amides do not form a nitrate in the presence of a hydroxyl radical scavenger. The *k*_NO3‑_ of a primary amide (i.e., acetamide) is quite low (≈0.1 M^–1^ s^–1^) and most of the amide bonds in NOM isolates are presumed to be proteinaceous (secondary amides).^20,60^ Therefore, the nitrate formed during ozonation of NOM isolates in the presence of a hydroxyl radical scavenger is likely not derived from amide moieties.

#### 3.3.3 δ^15^N in Nitrate Formed during Ozonation of NOM Isolates

Figure 3c,d shows the nitrogen isotope fractionation in nitrate formed during ozonation of the NOM isolates. Both SRNOM and UMRNOM exhibit two phases, identified based on the results of the nitrate formation assay, each with different nitrogen isotope fractionations. For SRNOM (Figure 3c), amino acids and peptidic amino groups were converted to nitrate first, accounting for approximately 30% of the total nitrate concentration formed during the nitrate formation assay, with an ε_N_ of -24.1 ± 2.8‰. Ensuing nitrate formation from aliphatic and aryl-type primary amine moieties showed a smaller nitrogen isotope fractionation (ε_N_ = -8.6 ± 1.5‰). Due to their similar ε_N_ values and simultaneous nitrate formation under the applied ozone exposure, aliphatic and aryl-type primary amine moieties could not be further distinguished using this method. Similarly, for UMRNOM (Figure 3d), 37% of the total formed nitrate displayed an enrichment factor (ε_N_) of -22.5 ± 1.6‰ (amino acids or peptidic amino groups), followed by -9.5 ± 1.3‰ for nitrate generated from aliphatic and aryl-type primary amine moieties.

#### 3.3.4 Overall Evaluation of Nitrogenous Moieties in NOM Isolates

The two oxidative assays using chlorine and ozone provide complementary qualitative and quantitative information about amine moieties in model compounds and DOM. The chloramine formation assay requires only a single reaction step. It quantifies the total reactive amine concentration and differentiates between various amine moieties, such as secondary and primary amines and amino acids. However, it does not distinguish peptidic amino groups from primary amines. In contrast, the nitrate formation kinetics assay involves multiple elementary reactions, leading to nitrate formation. However, due to the low nitrate formation rate constants of certain amines (e.g., secondary and aliphatic primary amines), this assay does not allow for the determination of total reactive amine concentrations. Instead, it provides insights into the differentiation of amino acids and peptidic amino groups from secondary and primary amines. Furthermore, it enables the distinction between aryl-type primary amines and primary and secondary aliphatic amines.

Figure 5 and Table S6 show the relative distribution of reactive amine moieties quantified by the different assays for SRNOM and UMRNOM. Fractions of total chloramine-forming amine moieties for standard NOM isolates were approximately 23–27% of DON, with 5–6%, 14–20%, and 1–4% for secondary amines, primary amines, and amino acids, respectively (Figure 5 and Table S6). The fractions of nitrate-forming amine moieties for standard NOM isolates were about 10–11% of DON in total, with 3%, 4%, and 3–4% as secondary and aliphatic primary amine, aryl-type primary amine, and amino acids and peptidic amino groups, respectively (Figure 5 and Table S6). The fractions of nitrate-forming amine moieties were significantly lower than the fraction of chloramine-forming amine moieties due to the low nitrate formation rate constants for secondary and aliphatic primary amines (e.g., *k*_NO_3_‑_ of ethylamine <0.1 M^–1^ s^–1^). These amines may not be fully converted to nitrate during ozonation. Therefore, the difference between the two assays (i.e., 17% for SRNOM (27–10)% and 12% for UMRNOM (23–11)%, respectively, Figure 5) is attributed to incomplete conversion of secondary and aliphatic primary amines in the nitrate kinetics assay. The fraction of amino acids estimated in the chloramine formation assay was smaller than that determined within the nitrate formation kinetics assay. This difference arises because peptidic amino groups behave like aliphatic or aryl-type primary amines in the chloramine formation assay but resemble amino acids in the nitrate formation kinetics assay. The difference for SRNOM (i.e., 2% for SRNOM (3–1)% (Figure 5) is attributable to peptidic amino groups. For UMRNOM, the difference is negligible, indicating a minimal fraction of peptidic amino groups. The fractions of nitrate-forming amine moieties were confirmed by SIA of nitrate, showing that 3–4% of the DON in SRNOM and UMRNOM could be attributed to amino acids and peptidic amino moieties, while the remaining 7% was linked to secondary amines, aliphatic primary amine, and aryl-type primary amine moieties (Table S6). The ε_N_ values of amino acid and peptidic amine moieties are very similar and cannot be distinguished using SIA. The same applies to the secondary amines, aliphatic primary amines, and aryl-type primary amine moieties. Overall, the relative composition of amine moieties in SRNOM and UMRNOM appears similar despite the different organic matter sources. However, there might be larger differences in the *N* composition and functional groups for other NOM sources (e.g., wastewater effluents, algal bloom-derived water, surface water, etc.),^22^ which warrants further studies. Fulvic acids were not included in this final assessment because of their limited extent of nitrate formation (e.g., maximum <1.5 μM at an ozone exposure of 1 Ms for a DOC concentration of ∼ 24 mgC/L) (Figure S19a), which is insufficient to perform stable isotope analyses.

**Figure 5:**
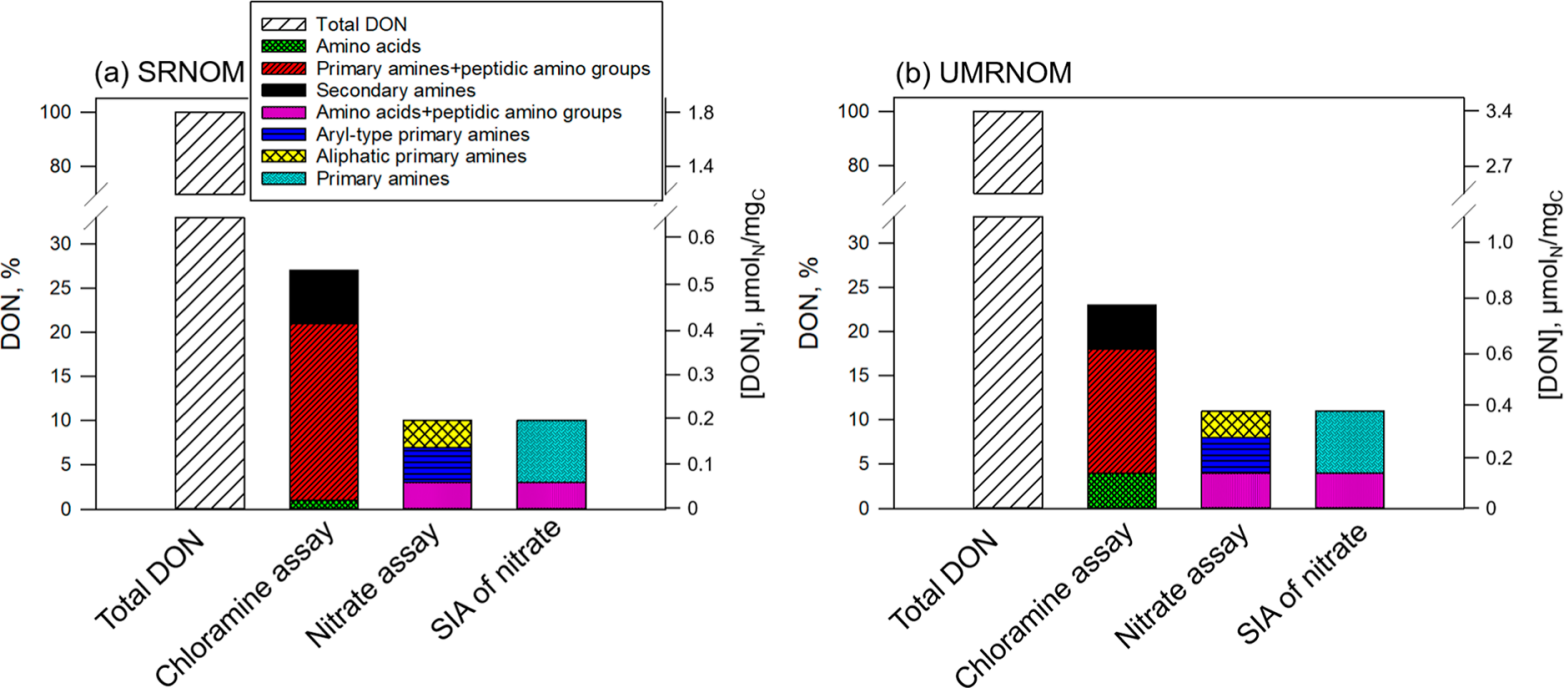
Distribution of amine moieties in (a) SRNOM and (b) UMRNOM, quantified by the chloramine and nitrate formation kinetics assays in percentage of the estimated DON. SIA of nitrate was used to assess if the nitrate is formed from amine moieties of amino acids (or peptidic amino groups) or primary amines. Care should be taken when comparing results of both techniques, as the nitrate assay and SIA of nitrate do not quantitatively capture secondary and aliphatic primary amines and therefore underestimate the total amine concentrations. [DON] was calculated based on the DON content (1.8 and 3.4 μmolN/mgC for SRNOM and UMRNOM, respectively) multiplied by DON % of each amine moiety. Note that the unit scale of [DON] for SRNOM is approximately a factor of 2 smaller compared to UMRNOM. Total DON denotes total dissolved organic nitrogen estimated from the elemental nitrogen-to-carbon ratio (N/C) of NOM isolates (Table S5),^45^ and primary amines include aliphatic and aryl-type primary amines.

### 3.4 Practical Implications

Oxidant-reactive amines are electron-rich moieties and likely serve as major precursors for *N*-DBPs during oxidative water treatment. Using the characterization results of oxidant-reactive amine moieties from a combination of three complementary approaches, the formation potential of four representative *N*-DBPs (total nitroalkanes, chloropicrin, dichloroacetonitrile, and *N*-nitrosodimethylamine) was estimated under oxidative water treatment processes (Table 2). The concentrations of different types of reactive amine moieties, which serve as precursors of *N*-DBPs, were determined from the findings in Section 3.3.4. As a reference, the formation yields of four representative *N*-DBPs for four oxidative water treatment processes were obtained from previous studies.^26,27,61–65^

**Table 2:** Estimated *N*-DBPs Formation Potentials for Different Water Treatments with DON Contents of NOM Isolates (=2.6 μmol/mgC) Used in This Study and in Previous Studies

*N*-DBPs	treatment leading to N-DBP formation	[precursor]_0_μmol/mgC	formation yield % (model compounds)	[*N*-DBPs] estimated, this study; [*N*-DBPs] measured, previous study, nmol/mgC
total nitroalkanes	ozone	0.6^a^	10–100 (glycine and ethylamine)^26,64,65^	400–500; N.A.^e^
chloropicrin	ozone followed by chlorine	0.4–0.5^b^	1.3 (Boc-lysine)^26^	5–7; 4–6 ^26^
dichloro-acetonitrile	chlorine	0.5^c^	1.2–6.0 (aspartic acid)^61,62^	6–30; 12–15 ^66,67^
*N*-nitrosodimethylamine	chloramine	0.2^d^	2.0 (dimethylamine)^27,63^	4; 0.1–0.5 ^19,68,69^

a Secondary amines + aliphatic and aryl type-primary amines + amino acids and peptidic amino groups (25% of DON, average from SRNOM and UMRNOM in Table S6).

b Total nitroalkanes estimated in Table 2.

c Aliphatic and aryl type-primary amines + amino acids and peptidic amino groups (20% of DON, average from SRNOM and UMRNOM in Table S6).

d Secondary amines (6% of DON, average from SRNOM and UMRNOM in Table S6), all obtained by calculating the average of SRNOM and UMRNOM.

e Not available.

A complete oxidation of all reactive amine moieties to nitrate in NOM is not expected for typically applied specific ozone doses in water treatment (examples of ozone exposures: ∼ 0.02 Ms for 1.0 gO_3_/gDOC in drinking water;^70^ ∼ 0.003 Ms for 0.6 gO_3_/gDOC in secondary wastewater effluent^71^). Instead, nitroalkanes might be formed at typical ozone doses. Given the formation yield of nitroalkane from amino acids (20%),^26,65^ primary amines (100%),^26,50^ and secondary amines (10–80%),^64^ ∼ 0.4 to 0.5 μmol/mgC of total nitroalkanes could potentially be formed from all types of reactive amine moieties in the standard NOM isolates (0.6 μmol/mgC, Table 2). Since no experimental studies are available that investigate the formation of total nitroalkanes during the ozonation of natural water or NOM isolates, a comparison with the estimated value was not made. The formed nitroalkane during ozonation of reactive amine moieties become potential precursors of halonitroalkanes during subsequent chlorination.^6,26,62,64^ Based on the chloropicrin formation potential (∼1.3%) observed from the chlorination of tertiary butyloxylcarbonyl (Boc)-protected nitrolysine over 3 days,^26^ ∼ 5–7 nmol/mgC of chloropicrin could potentially be formed from the chlorination of the totally formed nitroalkanes after ozonation of the standard NOM isolates (Table 2). This is in agreement with a previous finding for ozonation followed by chlorination of SRNOM isolates and urban stream water followed by chlorination.^26^ Nevertheless, under realistic conditions, the formation of chloropicrin can be mitigated by a biological post-treatment before chlorination because nitromethane, its potential precursor, is biodegradable.^53,72^ For chlorine disinfection (∼1 mgCl_2_/L), the primary amine moieties in the standard NOM isolates are all converted to monochloramine, with consecutive dichloramine formation. Dichloramines are decomposed to nitriles and become potential precursors of dichloroacetonitrile.^6,28^ Taking into account a formation yield of dichloroacetonitrile of about 1–6% based on formation potential tests during chlorination of aspartic acid (for ≤1 day),^61,62^ ∼ 6–30 nmol/mgC of dichloroacetonitrile could potentially be formed from aliphatic and aryl-type primary amines, amino acids, and peptidic amino groups in the standard NOM isolates (Table 2). Our estimate is in agreement with previous findings for chlorination of SRNOM isolates and surface water (12–15 nmol/mg, Table 2).^66,67^ During chloramination, *N*-nitrosodimethylamine (NDMA) is formed from the reaction with secondary amine moieties in NOM.^6,19^ Considering typical formation potentials of NDMA from dimethylamine (∼2%) during chloramination,^27,63^ ∼4 nmol/mgC of NDMA is expected to be formed from secondary amine moieties in the standard NOM isolates if we assume dimethylamine as the main representative of this group. The estimated NDMA formation potential obtained from standard NOM isolates in this study is ≤40 times higher compared to NDMA formation potentials in SRNOM isolates and real water samples observed in previous studies (Table 2).^19,68,69^ This large difference can be attributed to the assumption that dimethylamine is the main secondary amine moiety in NOM isolates.^68^ However, this is unrealistic and probably the reason for the overestimated NDMA formation potential. Overall, the formation potential of *N*-DBPs upon individual oxidative water treatments of DOM could be estimated within certain boundaries, based on the quantitative information on reactive amine moieties in DOM derived from the novel approaches in this study.

## Supplementary Material



## Notes

Reagents, details of chloramine formation assay and continuous ozonation, analytical approach for the determination of δ^15^N in nitrate, and application of three complementary assays to natural organic matter experiments (PDF)
