# Supplementary material for: Characterization of Organic Nitrogen by Chlorination, Ozonation, and Stable Isotope Analysis of Nitrate
Source: Environ Sci Technol. 2025 Jun 24;59(26):13481–93. doi: 10.1021/acs.est.5c01034 (PMC12243089; doi:10.1021/acs.est.5c01034)
Supplement: Supplementary file 1 [file es5c01034_si_001.pdf]

Supporting Information

Characterization of organic nitrogen by chlorination, ozonation, and stable isotope analysis of nitrate

Jiwoon Ra[a], Kun Huang[b], Joachim Mohn[b], Thomas B. Hofstetter[a,c], Elisabeth Muck[a], Urs von Gunten[a,d]*

[a]*Eawag, Swiss Federal Institute of Aquatic Science and Technology, CH-8600 Dübendorf, Switzerland*

[b]*Empa, Swiss Federal Laboratories for Materials Science and Technology, CH-8600 Dübendorf, Switzerland*

[c]*Institute of Biogeochemistry and Pollutant Dynamics (IBP), ETH Zürich, 8092 Zürich, Switzerland*

[d]*School of Architecture, Civil, and Environmental Engineering, École Polytechnique Fédérale de Lausanne, 1015 Lausanne, Switzerland*

*Corresponding author

Mailing address: Department of Water Resources & Drinking Water, Eawag, Swiss Federal Institute of Aquatic Science and Technology, CH-8600 Dübendorf, Switzerland

Phone: 41-58-765-5270, fax: 41-58-765-5802, email: urs.vongunten@eawag.ch

Submitted to Environmental Science & Technology

This SI includes 39 pages, 8 Texts, 6 Tables, and 20 Figures.

Table S1. List of the selected chemicals and reagents with the supplier and the purities

Compounds	Supplier	Purity	CAS-Number
Model *N*-compounds			
Diethylamine hydrochloride	Sigma-Aldrich	98%	660-68-4
N-Benzylmethylamine	Sigma-Aldrich	97%	103-67-3
Glycine	Sigma-Aldrich	99%	56-40-6
Phenylalanine	Sigma-Aldrich	99%	63-91-2
Alanine	Sigma-Aldrich	98%	56-41-7
Asparagine	Sigma-Aldrich	≥98%	5794-13-8
Aspartic acid	Sigma-Aldrich	99%	56-84-8
2-Allylglycine	Sigma-Aldrich	98%	7685-44-1
Tryptophan	Sigma-Aldrich	≥98%	73-22-3
Tyrosine	Sigma-Aldrich	98%	60-18-4
Lysine	Sigma-Aldrich	≥98%	657-27-2
Gly-gly	Sigma-Aldrich	99%	556-50-3
Gly×5	Sigma-Aldrich	N.A [a]	7093-67-6
Ethylamine solution	Sigma-Aldrich	66–72%	75-04-7
β-alanine	Sigma-Aldrich	99%	107-95-9
γ-aminobutyric acid	Sigma-Aldrich	99%	56-12-2
Gabapentin	Supelco	N.A.	60142-96-3
Benzylamine	Sigma-Aldrich	99%	100-46-9
p-chlorobenzylamine	Sigma-Aldrich	98%	104-86-9
Nitroethane	Alfa Aesar	99%	79-24-3
Nitromethylbenzene	Sigma-Aldrich	95%	622-42-4
Acetaldoxime	Alfa Aesar	98%	107-29-9
NOM isolates			
Suwannee River NOM (SRNOM)	International Humic Substances Society (IHSS)	N.A.	N.A.
Upper Mississippi River NOM (UMRNOM)	IHSS	N.A.	N.A.
Suwannee River Fulvic Acid (SRFA)	IHSS	N.A.	N.A.
Nordic Lake Fulvic Acid (NLFA)	IHSS	N.A.	N.A.
Nitrate standard			
USGS 32, 34 (potassium nitrate)	United States Geological Survey (USGS)	N.A.	7757-79-1
USGS 35 (sodium nitrate)	USGS	N.A.	7631-99-4
S1,S2, and S6 (ammonium	In-house standard[1]	N.A.	6484-52-2

nitrate)			
Other compounds/reagents (e.g., buffer, quenchers, etc.)			
tertiary-Butanol (*t*-BuOH)	Sigma-Aldrich	≥99.7%	75-65-0
Sodium hypochlorite solution	Sigma-Aldrich	10-15% (aq.)	7681-52-9
3-Buten-2-ol	Sigma-Aldrich	97%	598-32-3
Sodium phosphate monobasic monohydrate	Sigma-Aldrich	≥99.0%	10049-21-5
Sodium phosphate dibasic dihydrate	Sigma-Aldrich	≥98.0%	10028-24-7
Sodium tetraborate decahydrate	Sigma-Aldrich	≥99.5%	1303-96-4
Boric acid	Fluka	≥99.5%	10043-35-3
Sodium thiosulfate pentahydrate	Merck	99.5-100%	10102-17-7
N,N-Diethyl-*p*-phenylenediamine sulfate salt (DPD)	Fluka	≥98%	6283-63-2
Potassium iodide (KI)	Sigma-Aldrich	≥99%	7681-11-0
Resorcinol	Sigma-Aldrich	≥98%	108-46-3
Titanium(III) chloride solution	Sigma-Aldrich	10-15% (aq.)	7705-07-9
HCl	Sigma-Aldrich	10%	7647-01-0
Sulfamic acid	Sigma-Aldrich	99.3%	5329-14-6
Powdered activated carbon	Norit	N.A.	W15
Sodium chloride	Sigma-Aldrich	≥99.5%	7647-14-5

N.A.: Not available

Text S1. Selection of model *N*-compounds

The following 22 model *N*-compounds were selected in this study: 2 aliphatic or aryl-type secondary amines (diethylamine and *N*-benzylmethylamine), 9 amino acids (glycine, phenylalanine, alanine, asparagine, aspartic acid, 2-allylglycine, tryptophan, tyrosine, and lysine), 2 polypeptides (gly-gly and gly-gly-gly-gly-gly), 4 aliphatic primary amines (ethylamine, β-alanine, γ-aminobutyric acid, and gabapentin), 2 aryl-type primary amines (benzylamine and *p*-chlorobenzylamine), 2 nitroalkanes (nitroethane and nitromethylbenzene), and 1 oxime (acetaldoxime). The molecular structures of the model *N*-compounds are shown in Table 1. The information about the suppliers and purities of the reagents and the model *N*-compounds are specified in Table S1.

Text S2. Chloramine formation assay based on FAC/DPD/KI

Generally, primary and secondary amines have apparent second-order rate constants of 10^3–10^4 $M^{-1}s^{-1}$ for the reaction with HOCl at pH 6 to form monochloramine.[2] Monochloramines from primary amines can further react with HOCl to dichloramines with apparent second-order rate constants of ~10^2 $M^{-1}s^{-1}$ at pH 6.[2, 3] The stability of chloramines depends on the species of chloramines (i.e., monochloramine or dichloramine) and the structure of the precursors (i.e., the number and type of substituents). Monochloramonium derived from tertiary amine is extremely unstable with a half-life ($t_{1/2}$) of 2 min.[4] Monochloramines derived from secondary amines are quite stable with $t_{1/2}$ of tens of hours at pH 6.[5] Monochloramines derived from primary amines are stable, with $t_{1/2}$ ranging from a few hours to tens of hours, regardless of whether they are aliphatic (or aryl-type) primary amines or amino acids.[3, 6] Dichloramines derived from primary amines have highly variable $t_{1/2}$ depending on the structures of primary amines, with aliphatic (or aryl-type) primary amines being stable with $t_{1/2}$ of tens of hours [7], whereas chloro amino acids are quite unstable with $t_{1/2}$ on the order of 10 minutes.[6] Using these properties, chlorination offers the option to differentiate between aliphatic (or aryl-type) primary amines and amino acids.

The combined approach with diethyl-*p*-phenylene diamine (DPD)/potassium iodide (KI) is widely applied to quantify free available chlorine (FAC) and inorganic chloramines.[8] FAC reacts with DPD in absence of iodide. If low concentrations of KI (60 μM) are added, I_2/HOI is formed by reaction with monochloramine, which in turn reacts with excess DPD. Finally, if higher concentrations of KI (40 mM) are added, I_2 is formed from the reaction with dichloramines, with the ensuing reaction with excess DPD. The cumulative absorption profile of each sequential step indicates the concentrations of the individual oxidants (FAC, monochloramine, and dichloramine). In this study, the chloramine formation assay is adapted based on a protocol with DPD/KI, and we focus on organic chloramines.

Figure S1a and S2 shows a schematic representation of the experimental setup of the chloramine formation assay. To guarantee that all reactive amine compounds react with FAC to form monochloramine in a few seconds, excess of FAC (= 80 μM) was applied to the solution containing amine moieties. The reactions were performed at pH 6 to enable > 70% of monochloramine of the primary amines to react with FAC to dichloramine within 2 minutes. The experiments were performed in two sets, one for a reaction time of 2 min, and the other for a reaction time of 30 min. The former is to measure the total reactive amino compounds and the latter is to measure the total amine moieties without amino acids. After chlorination, 10 μM of resorcinol was added to quench the residual FAC for 1 min since the DPD cation radical is not stable for high absorption (e.g., $A_{510} > 0.8$). Note that the

apparent second-order rate constant for the reaction of HOCl with resorcinol at pH 6 is about 10^3 $M^{-1}s^{-1}$, 5 orders of magnitude higher than for monochloramine.[9, 10] Due to the inconsistent residual concentration of FAC after chloramine formation across various samples and reaction durations, a standardized dose of resorcinol is necessary. The concentration of resorcinol (10 μM) was determined considering two standards: to fully quench residual chlorine within 1 min (chlorine demand of resorcinol ≥ 3 mol/mol)[10], and not to quench the formed I_2/HOI from the reaction of chloramines with KI. For example, the chloramine formation assay of ethylamine with 25 μM of resorcinol was not as precise as the experiment with 10 μM of resorcinol (data not shown) to quantify ethylamine because residual resorcinol could compete with DPD for I_2/HOI. Even in presence of NOM, there is no significant competition by 10 μM of resorcinol due to the high concentration of DPD (~2 mM), making this procedure suitable for NOM isolates. A 50 mM DPD solution was added (final concentration of 2 mM), which immediately represents the concentration of the residual FAC (A_1 or A_3 for reaction times of 2 min and 30 min, respectively). The next step is to add KI to the solution with a final concentration of 60 μM, which immediately indicates the concentrations of monochloramines of secondary amines (A_4). The absorbance A_4 was determined in two sets with reaction times of 2 min and 30 min, and the latter was used instead of the former to obtain a more precise concentration of secondary amines because monochloramine of primary amines could be still important at a reaction time of 2 min. The final step is an addition of 40 mM KI, which provides the total concentration of primary amines (A_2) for a reaction time of 2 min or total primary amines except amino acids (A_5) for a reaction time of 30 min. About 80 % of the dichloramines of amino acids are decomposed within 30 min. Therefore, the difference of A_2 and A_5 represents the estimated concentration of amino acids. For detailed examples of the chloramine formation assay for model *N*-compounds, see Table S4.

Among three types of amine moieties (primary, secondary amines, and amino acids), ammonium was interpreted as either a primary amine or an amino acid in the chloramine formation assay. During the chloramine formation assay optimized in this study, the removal rate of ammonium (i.e., NH_4-N 10 μM) due to the breakpoint chlorination was 60 % (data not shown) for a chlorine contact time of 30 minutes[11], which was the screening time for amino acids (unstable dichloramines). The remaining 40 % was accounted as primary amines (stable dichloramines). By taking the measured concentration of ammonium and its contribution to each type of amine moiety into consideration, the interference of ammonium could be corrected to quantify reactive organic amine moieties.

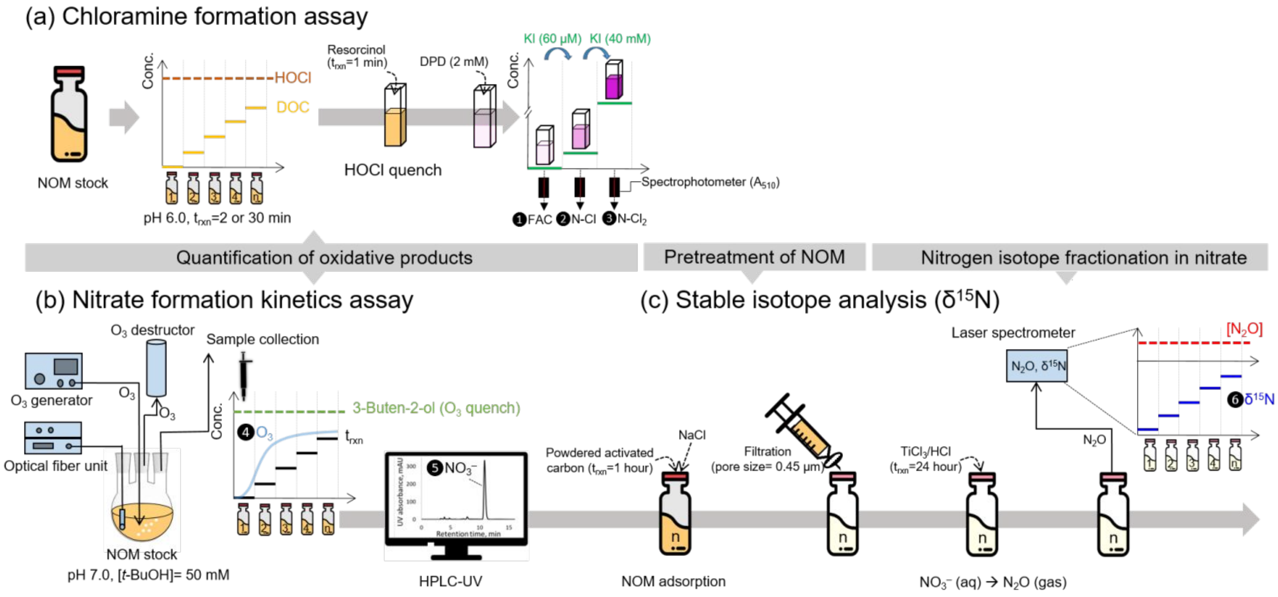

Figure S1. Schematic illustration of the experimental setups for the determination of amine moieties in NOM by (a) the chloramine formation assay, (b) the nitrate formation kinetics assay, and (c) stable isotope analysis. Monitoring parameters 1 to 3 (black numbered circles): concentrations of (1) FAC, (2) monochloramine, and (3) dichloramine spectrophotometrically quantified by DPD with sequentially increasing KI concentrations (lowest for FAC to highest for dichloramine), for increasing DOC concentrations (vials 1 to n) with fixed HOCl excess (= 80 μM), followed by addition of resorcinol. Monitoring parameter 4: the evolution of the ozone concentration during continuous ozonation quantified by an optical fiber (optical path length = 1 cm) in the range 220 to 400 nm. Monitoring parameter 5: the concentration of nitrate in collected samples (5 to 40 mL) quantified by HPLC-UV at 200 nm. Monitoring parameter 6: isotopic signature of $^{15}N/^{14}N$ ($\delta^{15}N$) in N_2O (from nitrate reduction), obtained by laser spectroscopy.

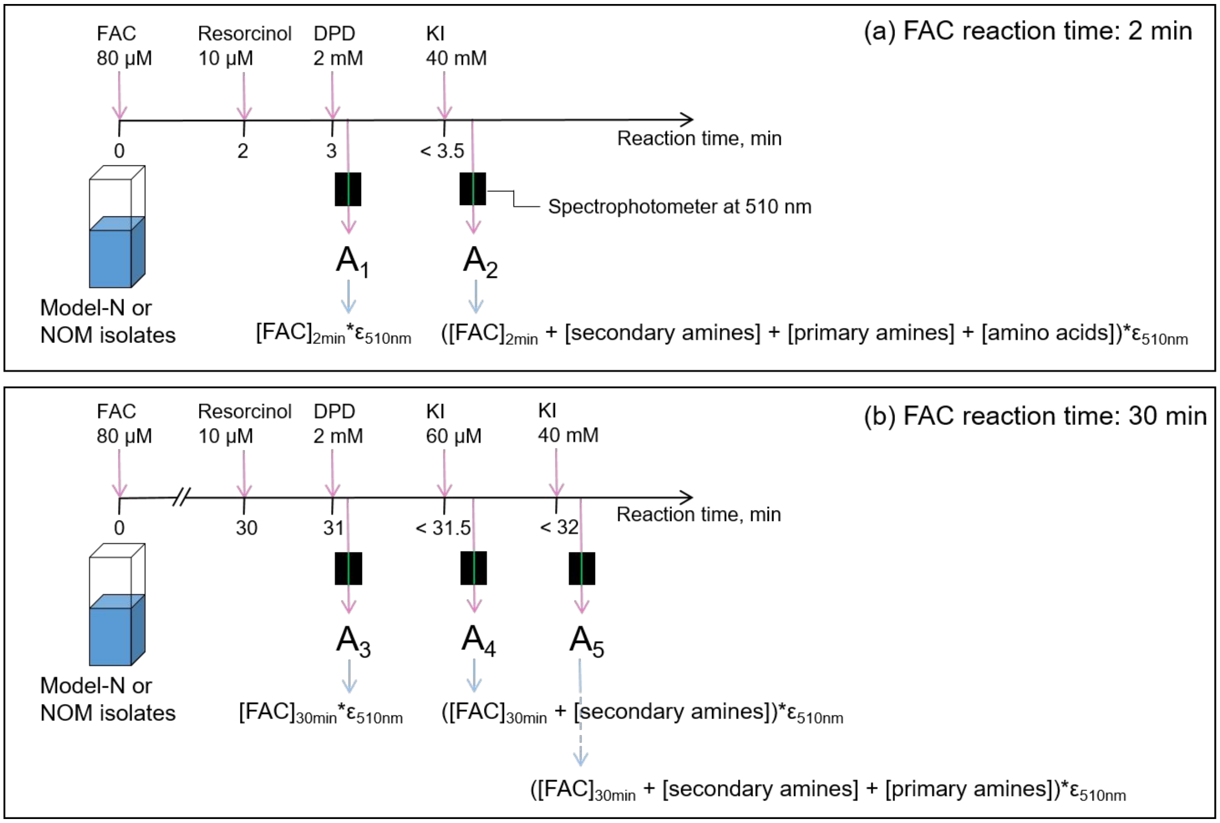

Figure S2. Schematic illustration of the experimental setup for the chloramine formation assay based on the chlorination of model *N*-compounds. The chlorination experiments were performed in two different reactors with the same mixtures but different chlorination time, (a) 2 minutes and (b) 30 minutes. Hereby, secondary amines, primary amines, and amino acids denote monochloramines, stable dichloramines, and unstable dichloramines, respectively. Monitoring parameters A_1 to A_5 at 510 nm: cumulative concentrations of residual free available chlorine (FAC) and combined chlorine were quantified by the DPD/KI method, with FAC for 2 min (A_1), FAC for 2 min + secondary amines + primary amines + amino acids (A_2), FAC for 30 min (A_3), FAC for 30 min + secondary amines (A_4), FAC for 30 min + secondary amines + primary amines (A_5). All the experiments were performed in 10 mM phosphate buffer at pH 6.

Text S3. Nitrate formation assay based on continuous ozonation

The scheme and setup for continuous ozonation is illustrated in Figures S1b and S3a. The ozone produced from an ozone generator with oxygen is sparged through a solution containing model *N*-compounds (or NOM isolates) in presence of 5 mM phosphate buffer, pH 7, and 50 mM *t*-BuOH (Figure S3b), which scavenges 99.9% and 98% of hydroxyl radical relative to model *N*-compounds (< 200 µM) and NOM isolates (< 50 mgC/L), respectively. An optical fiber (Avantes/AvaSpec-ULS2048CL-EVO) was immersed in the ozone reactor to monitor the real-time dissolved ozone concentration and to determine the ozone exposure (Figures S3b and S3c). The samples were collected at a sampling port of the reactor and immediately quenched with a 10-times molar excess of 3-buten-2-ol (relative to the ozone concentration). The presence of nitrite in the samples could affect isotopic analysis of N and O of nitrate in the next step. To mask the potentially formed nitrite resulting from the hydrolysis of partially oxidized nitrogenous compounds, sulfamic acid was added with a final concentration of 2 mM after quenching the residual ozone.[12] Finally, the nitrate samples were analyzed using High Performance Liquid Chromatography (Ultimate 3000, Dionex) coupled to a diode array detector (HPLC-UV, measuring wavelength 200 nm) with an anion-exchange column (IonPac AS9-HC, 4 mm×250 mm) under isocratic conditions with 40 mM sodium dihydrogen phosphate at 1 mL/min.

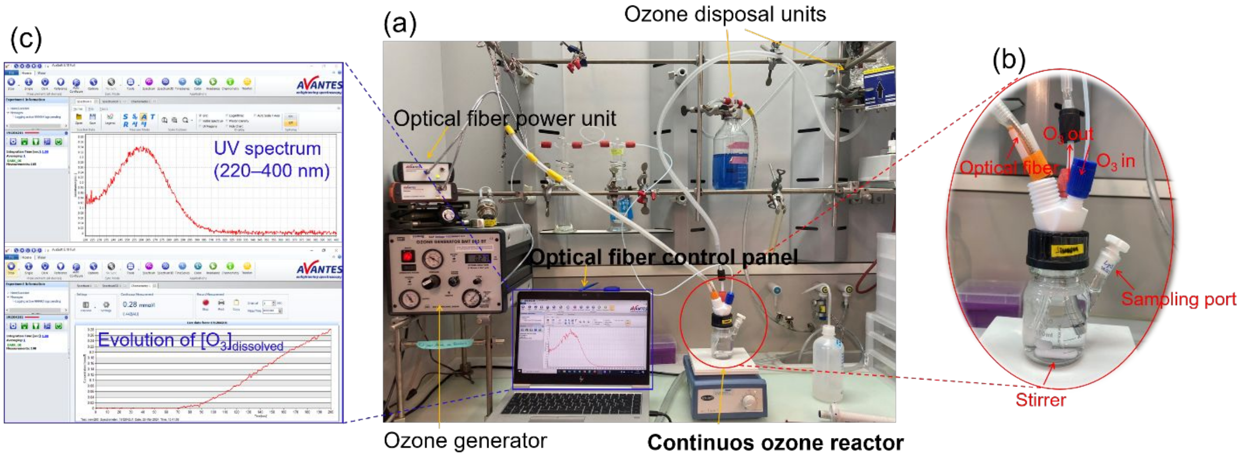

Figure S3. Experimental setup for continuous ozonation experiments. (a) Overall experimental setup including the ozone generator and the reaction vessel, (b) reaction vessel with optical fiber and sampling port and (c) ozone UV-Vis absorbance spectrum and evolution of the ozone concentration during ozonation measured by the optical fiber.

The concentration of dissolved ozone during continuous ozonation is adjusted depending on the temperature and ozone generator power, which controls the gas phase ozone concentration. For the compounds with high k_{NO3-} (e.g., > 20 $M^{-1}s^{-1}$), continuous ozonation was performed at room temperature (T= 293 K) and 8 % of the maximum ozone generator power to maintain a low dissolved ozone concentration (< 100 μM) for the initial 5–10 minutes. It allowed to collect at least 4 to 7 samples within 10 minutes before it was fully converted to nitrate. For the compounds with low k_{NO3-}, the ozone generator power was increased to 100 % to increase the dissolved ozone concentration (Figure S4). For complex compounds with multiple amine moieties (e.g., lysine), continuous ozonation experiments are typically performed with 8 % of the maximum ozone generator power for the initial 20 minutes, followed by an increase to 100 % to accelerate the conversion to nitrate.

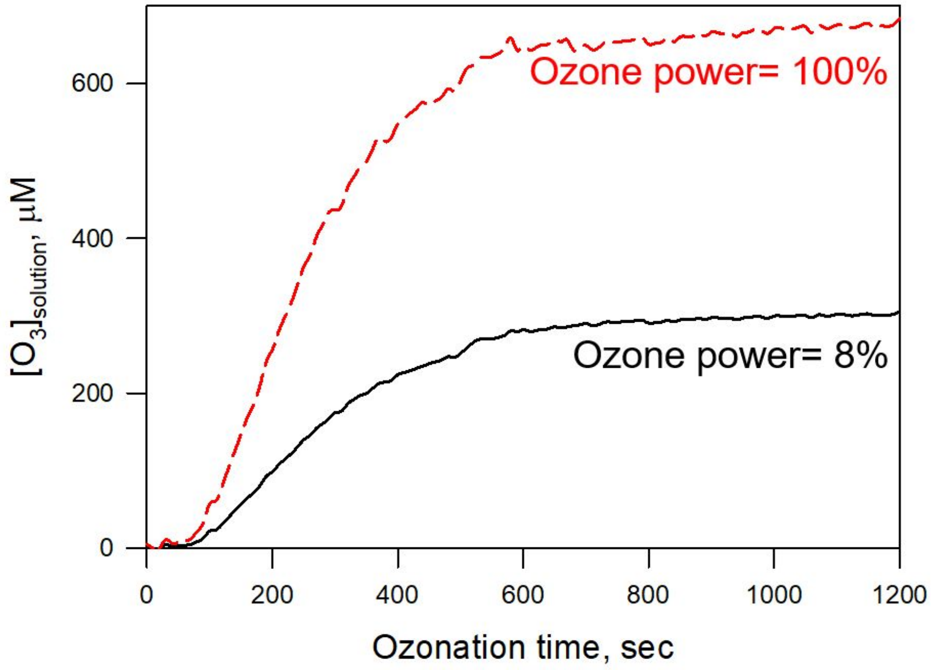

Figure S4. Evolution of dissolved ozone during continuous ozonation for different ozone generator powers (8 and 100 %). Experimental conditions: [glycine]= 20 μM, 50 mM *t*-BuOH, 5 mM phosphate, pH 7, T= 293 K.

During ozonation of NOM isolates, the measurement of dissolved ozone concentration is influenced by some fractions of the NOM isolate (e.g., phenol moieties), which interfere with the UV absorption of ozone, complicating the accurate determination of its concentration. To mitigate the interference of UV absorbance of the NOM matrix for ozone measurements (Figure S5a), the UV absorption was corrected at 260 nm by extrapolating the range of 320-400 nm, where dissolved ozone does not absorb

(Figure S5b). The change of the UV absorbance of NOM isolates during ozonation shows a linearity in the range of 320 – 400 nm, providing an option for the correction of the UV absorption by NOM at 260 nm. The slope of this linear correlation is an indication of the extent of change of the concentration of the phenolic moieties (Figure S6). For example, for an ozone exposure of 0.15 Ms the slope change increases in the order UMRNOM (0.001 $cm^{-1}nm^{-1}$, phenolic moiety concentration: 0.83 meq/g C) < SRNOM (0.002 $cm^{-1}nm^{-1}$, phenolic moiety concentration: 2.47 meq/g C) < SRFA (0.004 $cm^{-1}nm^{-1}$, phenolic moiety concentration: 3.11 meq/g C) < NLFA (0.0045 $cm^{-1}nm^{-1}$, phenolic moiety concentration: 3.18 meq/g C).[13,14]

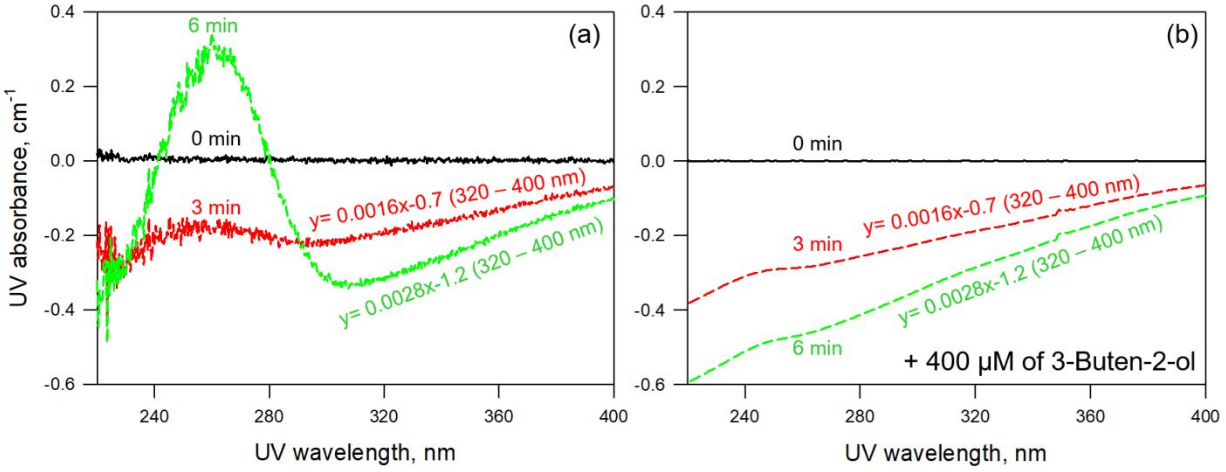

Figure S5. Continuous ozonation of SRNOM. (a) Evolution of UV spectra of the water matrix and (b) UV spectra of three samples after addition of 400 µM of 3-buten-2-ol at different ozonation times. Experimental conditions: ozone generator power = 8 % of maximum, [SRNOM] = 24 mgC/L, 50 mM *t*-BuOH, pH 7 (5 mM phosphate).

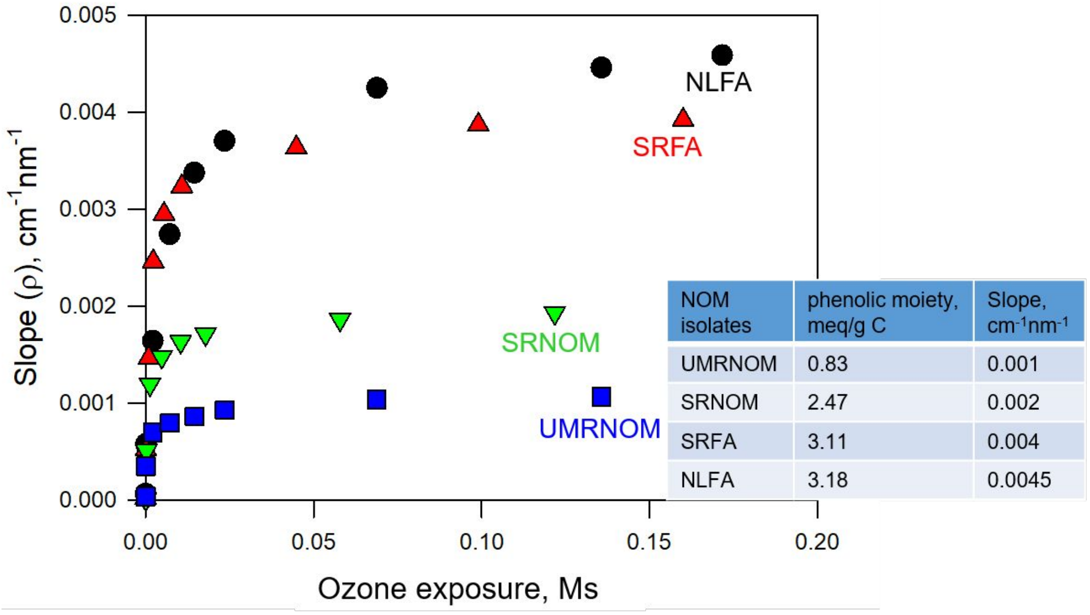

NOM isolates	phenolic moiety, meq/g C	Slope, $cm^{-1}nm^{-1}$
UMRNOM	0.83	0.001
SRNOM	2.47	0.002
SRFA	3.11	0.004
NLFA	3.18	0.0045

Figure S6. Evolution of the slope changes in UV-Vis spectra (320–400 nm) as a function of the ozone exposure during continuous ozonation of four NOM isolates. The inset table shows data for the concentrations of phenolic moieties in the NOM isolates (IHSS)[13] and the slopes of the UV spectra in the range of 320–400 nm at an ozone exposure of 0.15 Ms. Experimental conditions: ozone generator power = 8 % of maximum, [NOM isolates] = 20 mgC/L, 50 mM *t*-BuOH, pH 7 (5 mM phosphate).

The nitrate formation rate constants (k_{NO3-}) were obtained by eq. S1 based on eqs. 2 and 3 in the main manuscript.

$$k_{NO3-} = -\ln\,(1-[NO_3^-]/[\text{model } N]_0)\ /\ \int [O_3]dt \qquad \text{(S1)}$$

This equation can be rearranged to obtain absolute concentrations of nitrate as eq. S2.

$$[NO_3^-] = [\text{model } N]_0 \times (1-e^{-k_{NO_3^-}\int[O_3]dt}) \qquad \text{(S2)}$$

This equation was applied to model the measured nitrate formation as a function of ozone exposure in Figure 3. For lysine (Figure 3a) and a mixture of phenylalanine and p-chlorobenzylamine (Figure 3b), the fitting consists of two terms for the two moieties amino acids and aliphatic (or aryl-type) primary amines (eq. S3), which exhibits good correlations ($R^2 > 0.99$).

$[NO_3^-]_{total}$ = [amino acid]$_0\times(1-e^{-k_{NO_3^-,\text{ amino acid}}\int[O_3]dt})$ + [aliphatic (or aryl-type) primary amine]$_0\times$(1-

$e^{-k_{NO_3^-, \text{aliphatic (or aryl-type) primary amine}}\int[O_3]dt}$) (S3)

Because the initial concentrations of amino acids and aliphatic (or aryl-type) primary amines are known, k_{NO3-} values with limited uncertainty (up to ±2.3 $M^{-1}s^{-1}$) could be obtained. For NOM isolates (i.e., SRNOM and UMRNOM, Figures 3c and 3d), a fitting model with two terms shows unsatisfactory correlations ($R^2 = 0.94$), especially for the range of ozone exposures > 0.1 Ms ($R^2 < 0.9$) because there might be more than a single moiety in aliphatic (or aryl-type) primary amines. Thus, a fitting with three terms for amino acids, aryl-type primary amines, and aliphatic primary amines was tested as eq. S4, exhibiting good correlations ($R^2 > 0.99$).

$$[NO_3^-]_{total} = [\text{amino acid}]_0\times(1-e^{-k_{NO_3^-,\text{amino acid}}\int[O_3]dt}) + [\text{aryl-type primary amine}]_0\times(1-e^{-k_{NO_3^-, \text{aryl-type primary amine}}\int[O_3]dt}) + [\text{aliphatic primary amine}]_0\times(1-e^{-k_{NO_3^-, \text{aliphatic primary amine}}\int[O_3]dt}) \quad \text{(S4)}$$

Because the initial concentrations of individual reactive amine moieties in NOM isolates were not available, they were estimated simultaneously with k_{NO3-} values, resulting in a higher uncertainty (up to ±4.3 $M^{-1}s^{-1}$).

Text S4. General understanding of stable isotope ratio notation

Since isotope ratios (e.g., $^{15}N/^{14}N$ and $^{18}O/^{16}O$) and related changes are very small, they are typically provided relative to an isotope standard using the delta (δ) notation (eq. S5):

$$\delta X = ((X_{heavy}/X_{light})_{sample}/(X_{heavy}/X_{light})_{standard} - 1) \times 1000, ‰ \quad (S5)$$

where δX is the delta value of the nitrate sample for element X (N and O) in parts per thousand (permil, ‰) and X_{heavy}/X_{light} is the ratio of the heavy to light isotope in the nitrate sample and in an isotope standard, respectively.

Text S5. Analysis of N- and O- stable isotopic composition of nitrate

In this study, two different methodologies for SIA were applied for distinct purposes. Firstly, $^{15}N/^{14}N$ in nitrate samples were measured in commercial SIA laboratories using microbial transformation follwed by IRMS to screen ε_N during ozonation of model *N*-compounds. In addition, an alternative analytical procedures was developed in our laboratory using $TiCl_3$-based transformation followed by laser spectroscopy, which allowed on-site measurement of $^{15}N/^{14}N$ in nitrate samples, with a lower limit of quantification (1 μM instead of > 4 μM as NO_3^-).

Microbial transformation followed by measurement with IRMS

For the measurement of $^{15}N/^{14}N$ and $^{18}O/^{16}O$ in nitrate samples by commercial SIA laboratories, the nitrate samples were neutralized to pH 7 prior to transport. This was done to enable microbial transformation of nitrate to nitrous oxide. The $\delta^{15}N$ and $\delta^{18}O$ of nitrate samples were determined by commercial SIA laboratories at the University of California Davis, USA and the University of Calgary, Canada. The applied methods can be briefly summarized as follows[15, 16]: A 2-mL vial of denitrifying bacteria (i.e., *P. aureofaciens*) in soy broth, which is anaerobically grown and purged for up to 4 hours to remove N_2O, was added to a nitrate samples (up to 2 mL) and incubated overnight to allow complete conversion of NO_3^- to N_2O. Then, 0.2–0.3 mL NaOH was injected to lyse the bacteria. The formed N_2O was carried by helium to a gas chromatography-isotope ratio mass spectrometer (GC/IR-MS) coupled with a capillary column (30 m x 0.32 mm ID, 1.0 mL/min) to measure the oxygen and nitrogen isotopic composition. For calibration of the procedure, the certified nitrate reference standards, USGS32, USGS34, and USGS35 (Tables S1 and S3) were processed in every sample batch. The minimum concentration of nitrate to obtain $\delta^{15}N$ and $\delta^{18}O$ was 4 μM and the measurement errors were ± 0.4‰ for $\delta^{15}N$ and ± 0.5‰ for $\delta^{18}O$.

$TiCl_3$-based conversion followed by measurement with laser spectroscopy

To enhance the throughput for analysis of $\delta^{15}N$ in nitrate, an alternative approach was established involving a chemical reagent (titanium chloride, $TiCl_3$)[17] and a laser spectrometer. Two different sample preparation techniques were implemented (online and offline) with the potential to reduce measurement times to 8 min per sample for the online technique (details in Text S6 and Figure S7). For referencing of nitrate samples, six nitrate isotope standards were applied (Table S2, $\delta^{15}N$: -50.5 to

+180‰). The measurement error for $\delta^{15}N$ was in the range of ±0.8 to ±2.5‰ depending on the concentration of nitrate as well as the analytical method (Figures S8-S10). $\delta^{18}O$ analysis in nitrate with the $TiCl_3$-based approach was not possible, because of exchange of oxygen atoms with water during the conversion process of nitrate to N_2O. Overall, this allowed the measurement of low-concentration nitrate samples starting from 1 μM in NOM isolates, which would otherwise be impossible in the commercial SIA laboratories. Moreover, the analytical conditions in commercial SIA laboratories are not compatible with acidic pH (< 3) of the nitrate samples prepared in this study, which contain sulfamic acid. This incompatibility arises because under these conditions bacterial activity is compromised. Therefore, a new methodology involving a $TiCl_3$-based conversion to N_2O followed by spectroscopic analysis was developed and optimized (Text S6).

Text S6. Stable nitrogen isotope analysis in nitrate; applying a $TiCl_3$-based conversion to N_2O followed by spectroscopic analysis

The schematic illustration of stable isotope analysis of nitrogen ($\delta^{15}N$) in nitrate from ozonation of amine compounds is presented in Figure S1c. The $\delta^{15}N$ in nitrate were analyzed in a two-step procedure, first converting the dissolved nitrate to N_2O by reduction with $TiCl_3$ under acidic conditions (eq. S6), followed by N_2O analysis with an isotope specific laser spectrometer.[17]

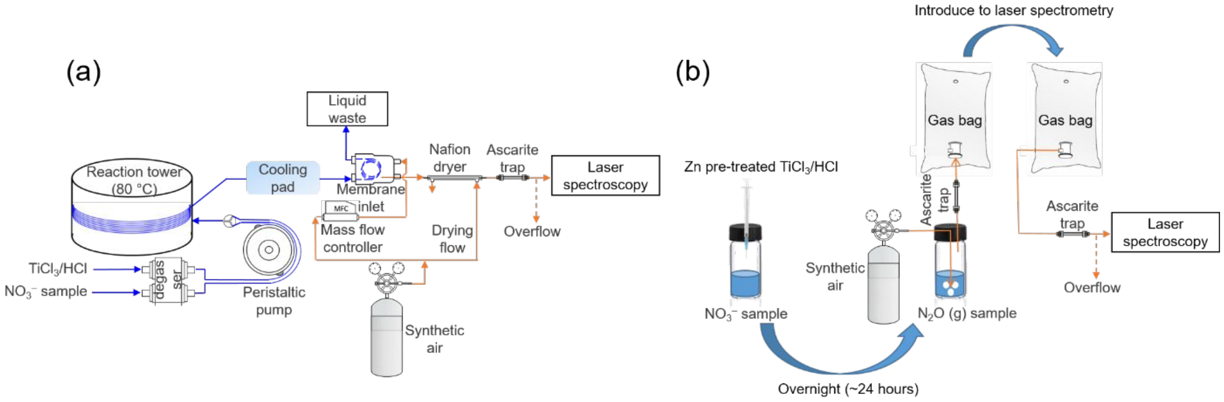

Figure S7. Detailed analytical setups for the measurement of $\delta^{15}N$ in nitrate via (a) online and (b) offline/batch conversion to N_2O prior to laser spectroscopic analysis.

Reduction of NO_3^- with $TiCl_3$ to N_2O

$$2\ NO_3^-{}_{(aq)} + 8\ Ti^{3+} + 10\ H^+ \rightarrow N_2O_{(g)} + 8\ Ti^{4+} + 5\ H_2O \qquad (S6)$$

Figure S7 shows the two approaches used for the conversion of nitrate, online conversion (Figure S7a) and offline/batch conversion (Figure S7b).

For the online conversion (Figure S7a), an automated system (named sample preparation unit for inorganic nitrogen species (SPIN)) was applied to achieve fast conversion at elevated temperatures (80 °C). This system was modified from a previous study that measured simultaneously the stable nitrogen isotopic compositions in ammonium, nitrate, and nitrite.[18] In the current system, samples or calibration solutions containing nitrate (50 – 200 μM nitrate; 1.0 mL min^{-1}) was mixed with the $TiCl_3$ reagent (approximately 150 mM; 1.2 mL min^{-1}) through a high pressure PEEK tee (1.59 mm × 1 mm, VICI AG International, Switzerland), and transported towards the reaction tower. The reaction tower was equipped with a fused silica coated PEEK capillary (0.53 mm I.D × 12 m length; BGB Analytik AG,

Switzerland), and heated to 80 °C using a polyimide thermofoil (475 mm × 43 mm, Thermo technologies, Germany). The system was automated with a customized LabVIEW code (National Instruments Corp., USA). In the reaction capillary, nitrate was converted to N_2O by reduction with $TiCl_3$ (eq. S6). The temperature of the reaction solution was lowered after the reaction tower to 25–30 °C with two cooling pads to protect the membrane inlet from damage (MINI Degassing Chamber, Systec AF, Sweden). Within the membrane inlet, the N_2O analyte permeated from the liquid phase into a flow of synthetic air carrier gas (20.5% O_2, 79.5% N_2, 99.999% purity; PanGas AG or Messer Schweiz AG, Switzerland). The synthetic air flow was adjusted with a mass flow controller (MFCs, 100 mL min^{-1}, Vögtlin Instruments, Switzerland), depending on the nitrate concentration (see discussion below). Downstream of the membrane inlet, the N_2O in the synthetic air mixture was first dehumidified with a Nafion dryer (MD-050, Perma Pure, USA, counterflow of 50 mL min^{-1} synthetic air), before residual traces of H_2O and CO_2 were removed with a $Mg(ClO_4)_2$/ascarite filled trap[18] Switching between N_2O sample and standard gases was accomplished with a gas selection valve with common outlet (VICI AG International, Switzerland). The N_2O isotopic composition was analysed by either QCLAS (named SPIN-QCLAS) or CRDS (named SPIN-CRDS) as described below.

For the offline/batch conversion (Figure S7b), a batch reactor containing nitrate and $TiCl_3$/HCl solution was incubated overnight (~ 24 h) to achieve complete conversion at room temperatures (T= 293 K). The batch conversion of nitrate to N_2O followed the approach established by a previous study.[17] The commercially acquired $TiCl_3$ solution (10-15%) was first pretreated with Zn powder, 2 h prior to use, to avoid potential perturbation by Ti^{4+}.[17] 0.9 mL pretreated $TiCl_3$ solution was then added to a 50 mL crimped bottle containing 20 mL sample and 0.2 mL HCl (10% v/v) using a syringe. The nitrate concentration of the samples was adjusted to 1–5 μM for improved reproducibility. Reaction bottles were kept in the dark for 24 h for complete conversion of NO_3^- to N_2O before the N_2O was purged into an aluminum coated gas bag with synthetic air at 20 mL min^{-1} for 12.5 min, resulting in a total gas volume of 250 mL, and the N_2O isotopic composition analyzed by CRDS. This approach is referred henceforth as batch conversion-CRDS.

N_2O isotopic analysis by laser spectroscopy

Two commercially available isotope specific N_2O analysers were used for the simultaneous analysis of the four most abundant N_2O isotopic species ($^{14}N^{14}N^{16}O$, $^{14}N^{15}N^{16}O$, $^{15}N^{14}N^{16}O$, and $^{14}N^{14}N^{18}O$) [19, 20], depending on the nitrate/N_2O concentrations. For model *N*-compounds of [model-*N*] = 250 μM (i.e., phenylalanine, alanine, and glycine), the formed nitrate was diluted to 50 μM and a quantum cascade

laser absorption spectroscopy (QCLAS; Aerodyne Research Inc., Billerica, USA) was used with a carrier gas flow of 8 mL min^{-1}. The spectrometer was equipped with a continuous-wave mid-infrared quantum cascade laser source (Alpes Lasers SA, Switzerland) emitting at 2203 cm^{-1}, and an astigmatic multi-pass absorption cell with a path length of 76 m and a volume of 0.62 L. Data acquisition and quantification of N_2O isotopocules was accomplished by the software TDL Wintel (Aerodyne Research Inc., Billerica, USA). For the rest of model *N*-compounds of [model-*N*] = 50 µM (or model *N*-compound with low $k_{NO3-} < 0.1$ $M^{-1}s^{-1}$ of [model- *N*] = 250 µM), formed nitrate was diluted to ≤ 5 µM and a more sensitive mid-infrared cavity ring-down spectrometer (CRDS; G5131-i, Picarro Inc., California, USA) was used, with a synthetic air carrier gas flow of around 20 mL min^{-1}. In CRDS, laser light of a single-frequency continuous wave laser diode (2195.7-2196.3 cm^{-1}) enters a three-mirror cavity, resulting in an effective pathlength of over 8 km and the capability for highly sensitive analysis. A photodetector measures the decay of light inside the cavity after the laser diode is turned off to retrieve the mole fractions of individual N_2O isotopocules.

The performance of the analytical procedure was tested using four calibration gaseous N_2O (Cal 1 – Cal 4), as well as three international (USGS 32, USGS 34, USGS 35, Reston Stable Isotope Laboratory (RSIL), Reston, USA), and three laboratory-internal nitrate isotope standards (S1, S2 and S6) (see Table S2 for their isotopic composition).[1]

Table S2. $\delta^{15}N$ values of the applied calibration gaseous N_2O (Cal 1 – Cal 4) and nitrate isotope standards used in this study (all values are reported in ‰). The values of $\delta^{15}N^{\alpha}$ and $\delta^{15}N^{\beta}$ denote isotopic composition of the central $N^{*}NO$ and terminal $^{*}NNO$ N atoms in N_2O, respectively. The value of $\delta^{15}N$ represents the average of $\delta^{15}N^{\alpha}$ and $\delta^{15}N^{\beta}$.

	$\delta^{15}N^{\alpha}$	$\delta^{15}N^{\beta}$	$\delta^{15}N$
Cal 1 (N_2O) [a, c]	2.1 ± 0.5	2.0 ± 0.5	2.0 ± 0.2
Cal 2 (N_2O) [a, c]	-64.3 ± 0.5	-60.6 ± 0.5	-62.5 ± 0.2
Cal 3 (N_2O) [b, c]	51.0 ± 0.5	55.1 ± 0.5	53.0 ± 0.2
Cal 4 (N_2O) [b, c]	-66.4 ± 0.5	-62.7 ± 0.5	-64.5 ± 0.2
USGS 32 (nitrate)			+ 180 exactly
USGS 34 (nitrate)			−1.8 ± 0.1
USGS 35 (nitrate)			+ 2.7 ± 0.1
S1 (nitrate) [d]			−1.4 ± 0.1

S2 (nitrate) [d]			+13.7 ± 0.1
S6 (nitrate) [d]			−50.5 ± 0.1

[a] Applied for analysis using QCLAS

[b] Applied for analysis using CRDS

[c] Uncertainties are estimated based on previous studies[1, 21]

[d] Laboratory-internal nitrate isotope standards [1]

The isotopic composition of the N_2O analyte gases, generated from nitrate samples/standards was quantified by applying a two-point calibration approach with two pairs of gaseous N_2O isotope standards, referred to as Cal 1 & 2 and Cal 3 & 4 (Table S2). Both N_2O isotope calibration gases have been prepared in high purity synthetic air. Cal 1 and Cal 3 were also used as anchor gases, analyzed before and after each sample to monitor and if required correct drift effects of the laser spectrometers. In general, the CRDS analyzer did not exhibit significant drift effects during the analysis time, while the QCLAS instrument drifted over time and thus this effect was considered when calculating the $\delta^{15}N$-NO_3^-. For both analyzers, the values of $\delta^{15}N^{\alpha}$ ($^{14}N^{15}NO$) and $\delta^{15}N^{\beta}$ ($^{15}N^{14}NO$) were obtained individually based on calibration curves described by analysis of Cal 1 - Cal 4 (Table S2), and bulk $\delta^{15}N$ values were calculated as the average of $\delta^{15}N^{\alpha}$ and $\delta^{15}N^{\beta}$.

Validation of the analytical procedure

Using the SPIN-QCLAS procedure for analyzing $\delta^{15}N$-NO_3^- across different NO_3^- concentrations (10 – 200 μM), best agreement between measured and actual delta values was obtained at 50 μM NO_3^- with acceptable precision (standard deviation (SD) < 1.8‰) (Figure S8a). In addition, at 50 μM a linear response of measured versus actual delta values (‰) was observed, with an average offset of 0.93‰ (Figure S8b), across several NO_3^- isotope standards. Therefore, 50 μM NO_3^- was selected for later analysis by SPIN-QCLAS. The QCLAS output signal was averaged over 1 min, after an initial stabilization period (approximately 4 min). A measuring/averaging period longer than 1 min had no discernible effect on the measured values and standard deviations.

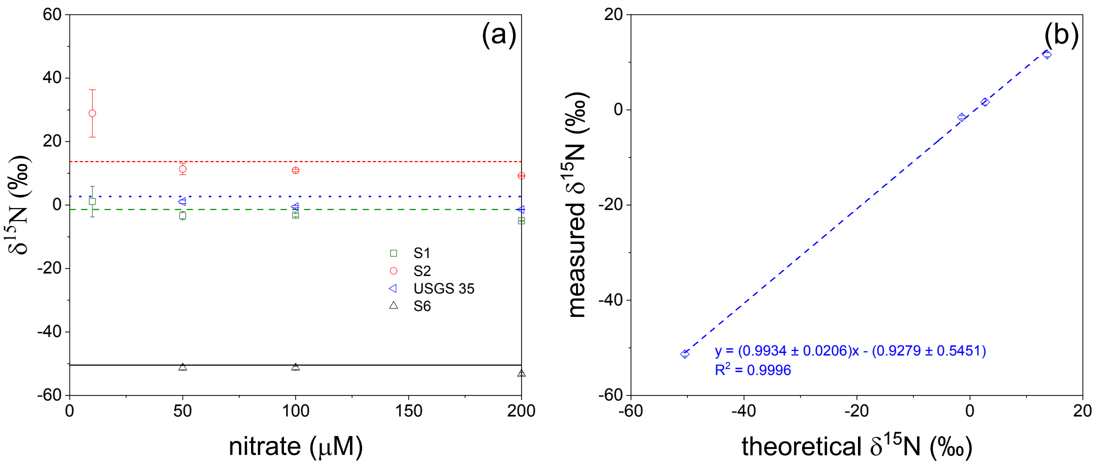

Figure S8. Effects of nitrate concentration on precision of $\delta^{15}N$ measurements applying SPIN-QCLAS. (a) $\delta^{15}N$ measurements at 10 – 200 μM NO_3^-, (b) comparison of measured and actual $\delta^{15}N$ values at 50 μM NO_3^- using S1, S2, S6, USGS 35.

SPIN-CRDS was used for nitrate concentration ≤ 5 μM, and similar validation experiments were conducted to check the system performance (Figure S9). Standard deviations were obtained from the averaged CRDS output signals over 2 min after an initial stabilization period. For the tested concentration range (3 – 6 μM nitrate), a larger offset (7.6‰) and SD (up to 2.4‰) was observed at 3 μM, while best performance was achieved at 6 μM with an offset of 0.9‰ and SD < 0.8‰, slightly better than at 5 μM (offset = 1.6‰, SD < 0.8‰).

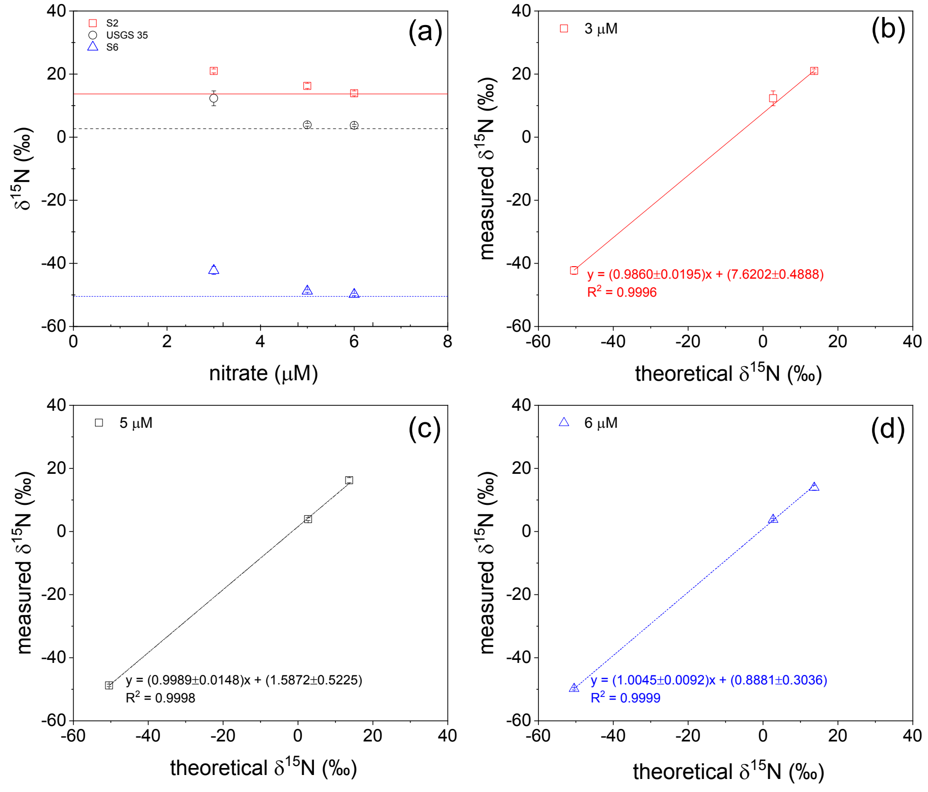

Figure S9. Effects of nitrate concentration on precision of $\delta^{15}N$ measurements applying the SPIN-CRDS. (a) $\delta^{15}N$ measurements at 3 – 6 μM nitrate, and (b-d) comparison of measured and actual $\delta^{15}N$ values at 3 – 6 μM nitrate using S2, S6, and USGS 35, respectively.

As an alternative for low NO_3^- concentrations (< 5 μM), a second CRDS procedure after batch conversion at room temperature (batch conversion-CRDS) was developed (Figure S10). Analysis at 5μM and 1 μM NO_3^- showed considerable but constant offsets (-4.1‰ and -12.8‰) and acceptable SD of 0.6‰ and 0.9–2.5‰, respectively. For the experiments conducted here, 5 μM and 1 μM were selected to measure $\delta^{15}N$ values in nitrate formed from model *N*-compounds and NOM isolates, respectively, because of the ease of dilution to reach the target concentration. Depending on NO_3^- concentrations, delta values were corrected for offsets.

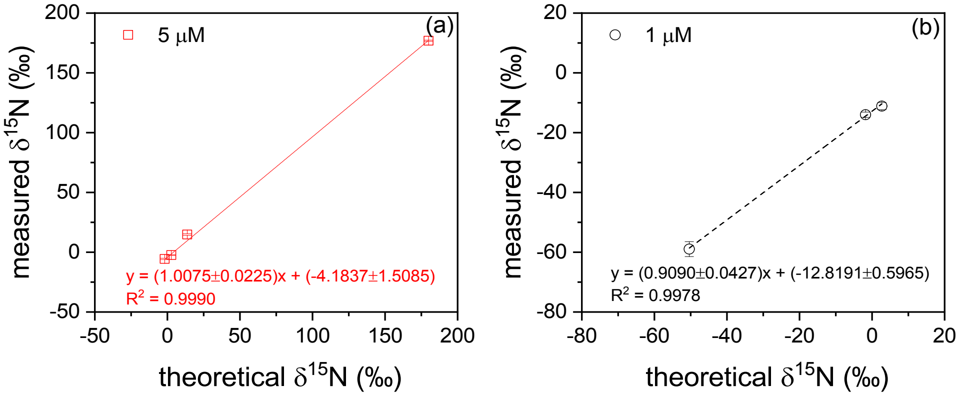

Figure S10. Comparison of measured and actual $\delta^{15}N$ at (a) 5 μM and (b) 1 μM nitrate using USGS 32, USGS 34, USGS 35 and S2 applying the batch conversion-CRDS. The standard deviations for 1 μM were obtained from measurements of three individual standards.

Table S3. $\delta^{15}N$ signatures in model *N*-compounds and NOM isolates used in this study. The values were obtained by an elemental analyzer isotope ratio mass spectrometer. Errors denote standard deviations of analytical triplicates.

Group	Compounds	$\delta^{15}N$, ‰
Model *N*-compounds	Ethylamine	N.D. [a]
	Nitroethane	-3.6±0.1
	Benzylamine	0.3±0.1
	Nitromethylbenzene	13.8±0.1
	p-chlorobenzylamine	4.5±0.1
	β-alanine	-2.5±0.1
	γ-aminobutyric acid	-8.3±0.2
	Gabapentin	-3.6±0.1
	Glycine	3.3±0.1
	Gly-gly	7.1±0.1
	Gly×5	N.D.[a]
	Phenylalanine	3.5±0.1
	Alanine	-1.5±0.1
	Asparagine	N.D.[a]
	Aspartic acid	0.1±0.1
	Acetaldoxime	-60.9±0.1
	Allylglycine	-49.3±0.3
	Tryptophan	N.D. [a]
	Tyrosine	N.D. [a]
	Lysine	0.1±0.1
NOM isolates	SRNOM	0.9±0.1
	UMRNOM	1.8±0.1
	SRFA	-2.8 [b]
	NLFA	-3.2 [b]

[a] Not determined, [b] determined by IHSS[13]

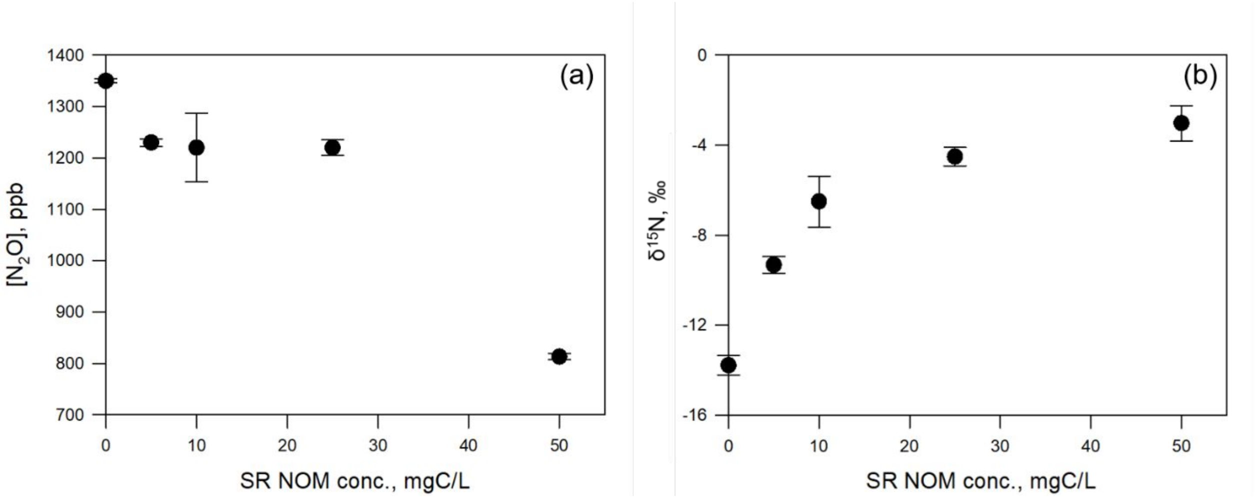

Figure S11. Nitrate conversion to N_2O by the $TiCl_3$ reagent in presence of SRNOM. Matrix effect of the SRNOM concentration (measured as DOC) on spiked nitrate standard (5 μM) for (a) $[N_2O]$ and (b) $\delta^{15}N$ measured by the batch conversion-CRDS at pH 7 (5 mM phosphate). Error bars denote standard deviations of analytical duplicates.

Text S7. Abatement of matrix effects in NOM isolates by powdered activated carbon

To minimize the matrix effect caused by NOM during $\delta^{15}N$ analysis of nitrate, it has to be removed from the solution before analysis. In this study powdered activated carbon (PAC) was selected (Figure S1c) because of its high adsorption efficiency (Figure S12). The removal efficiency for NOM was found to be proportional to the PAC dose, up to 85% removal at 3 g/L of PAC with a nitrate recovery efficiency of 95% in presence of 10 mM chloride, added to minimize nitrate adsorption (Figure S12). Overall, with our improved procedure, the initial 50 mgC/L NOM isolates used in this study were reduced to 7.5–10 mgC/L, enabling the measurement of N_2O concentrations and $\delta^{15}N$ of nitrate.

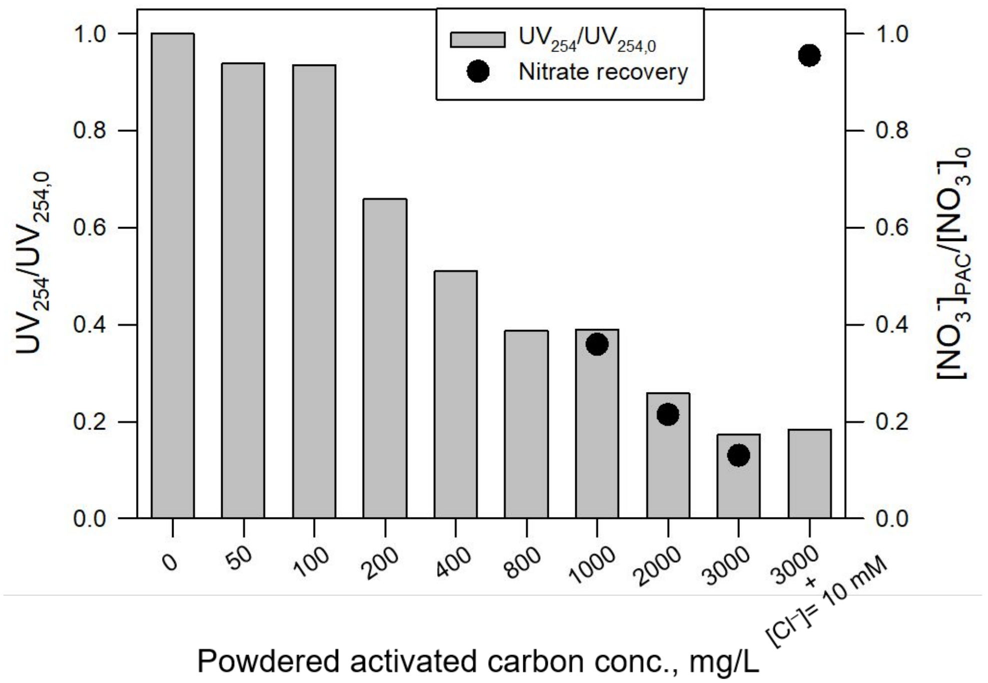

Figure S12. SRNOM removal by powdered activated carbon (PAC). Relative residual absorbance at 254 nm as a function of the PAC dose (grey bars) and the relative residual concentration of nitrate (black circles). $UV_{254,0}$ and UV_{254} denote absorbances at 254 nm before and after PAC treatment, respectively. $[NO_3^-]_0$ and $[NO_3^-]_{PAC}$ denote nitrate concentrations before and after PAC treatment, respectively. The relative residual nitrate concentration is shown with black circles. Experimental conditions: [SRNOM]= 50 mgC/L, $[NO_3^-]_0$= 10 µM, adsorption contact time 1 hour, pH 7 (5 mM phosphate).

Table S4. Results from colorimetric measurements during the chloramine formation assay for individual model *N*-compounds. All the absorbances (A_1 to A_5) were obtained based on the steps described in Figure S2. The molar absorption coefficient of the DPD cation radical at 510 nm (ε_{510}) is 19,800 $M^{-1}cm^{-1}$ [22]. Optical path length for absorbance measurements was 1 cm.

Model *N*-compounds		Obtained absorbances					Estimates of [amines]		
Group	[Model *N*]= 5 µM	A_1	A_2	A_3	A_4	A_5	secondary amines $(A_4-A_3)/\varepsilon_{510}$ [a]	Primary amines $(A_5-A_4)/\varepsilon_{510}$ [b]	Amino acids $((A_2-A_1)-(A_5-A_3))/\varepsilon_{510}$ [c]
Secondary amines	Diethylamine	0.17	0.28	0.23	0.33	0.34	5.0 µM	<0.5 µM	0 µM
	N-Benzyl methylamine	0.2	0.31	0.15	0.26	0.26	5.5 µM	0 µM	0 µM
Primary amines	Ethylamine	0.23	0.33	0.13	0.13	0.23	0 µM	5 µM	0 µM
	Benzylamine	0.21	0.32	0.17	0.18	0.28	<0.5 µM	5 µM	0 µM
Amino acids	Gly-gly [d]	0.19	0.31	0.24	0.25	0.36	<0.5 µM	5.5 µM	0 µM
	Glycine	0.23	0.34	0.09	0.09	0.1	0 µM	<0.5 µM	4.5 µM
	Phenylalanine	0.21	0.33	0.13	0.13	0.14	0 µM	<0.5 µM	5 µM

[a] determined [secondary amines], [b] determined [primary amines], [c] determined [amino acids], [d] peptidic amino groups are treated as primary amines in the chloramine formation assay due to their stability similar to dichloramine.

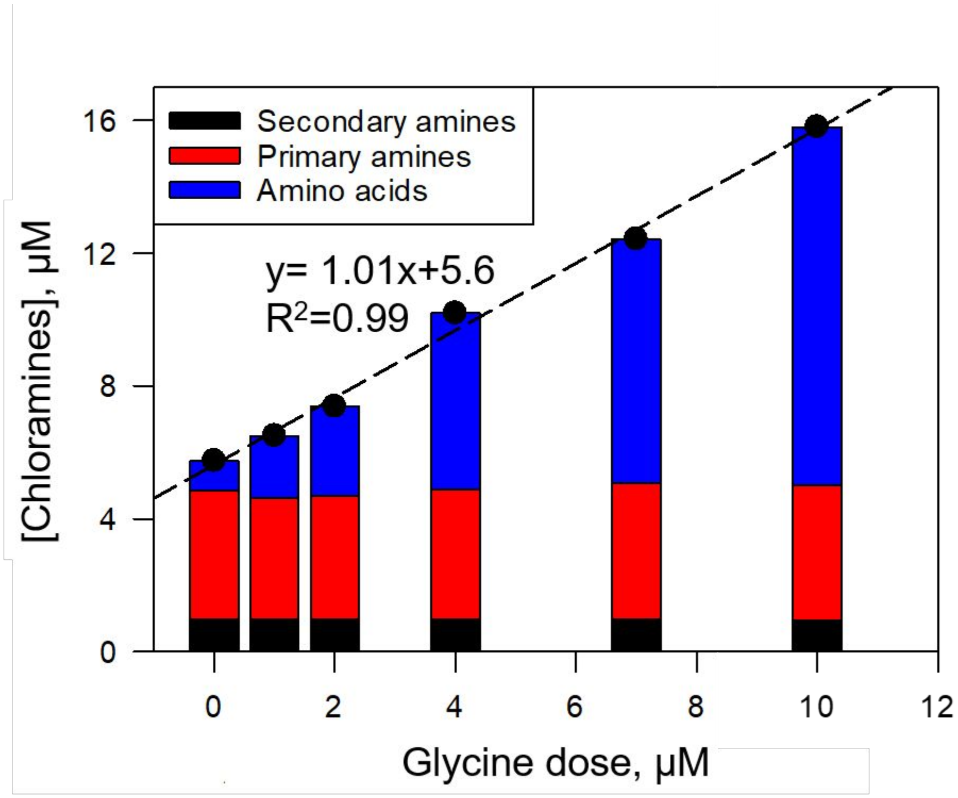

Figure S13. Screening of reactive amine moieties in 10 mgC/L SRNOM with varying glycine doses (0 – 10 µM) using the chloramine formation assay. Experimental conditions: [FAC] = 80 µM for 2 min and 30 min, followed by addition of [resorcinol] = 10 µM for 1 min, followed by [DPD] = 2 mM with [KI]= 0, 60 µM and 40 mM in sequence to measure [FAC], [*N*-Cl amine], and [*N*-Cl_2 amine], respectively, at pH 6 (10 mM phosphate).

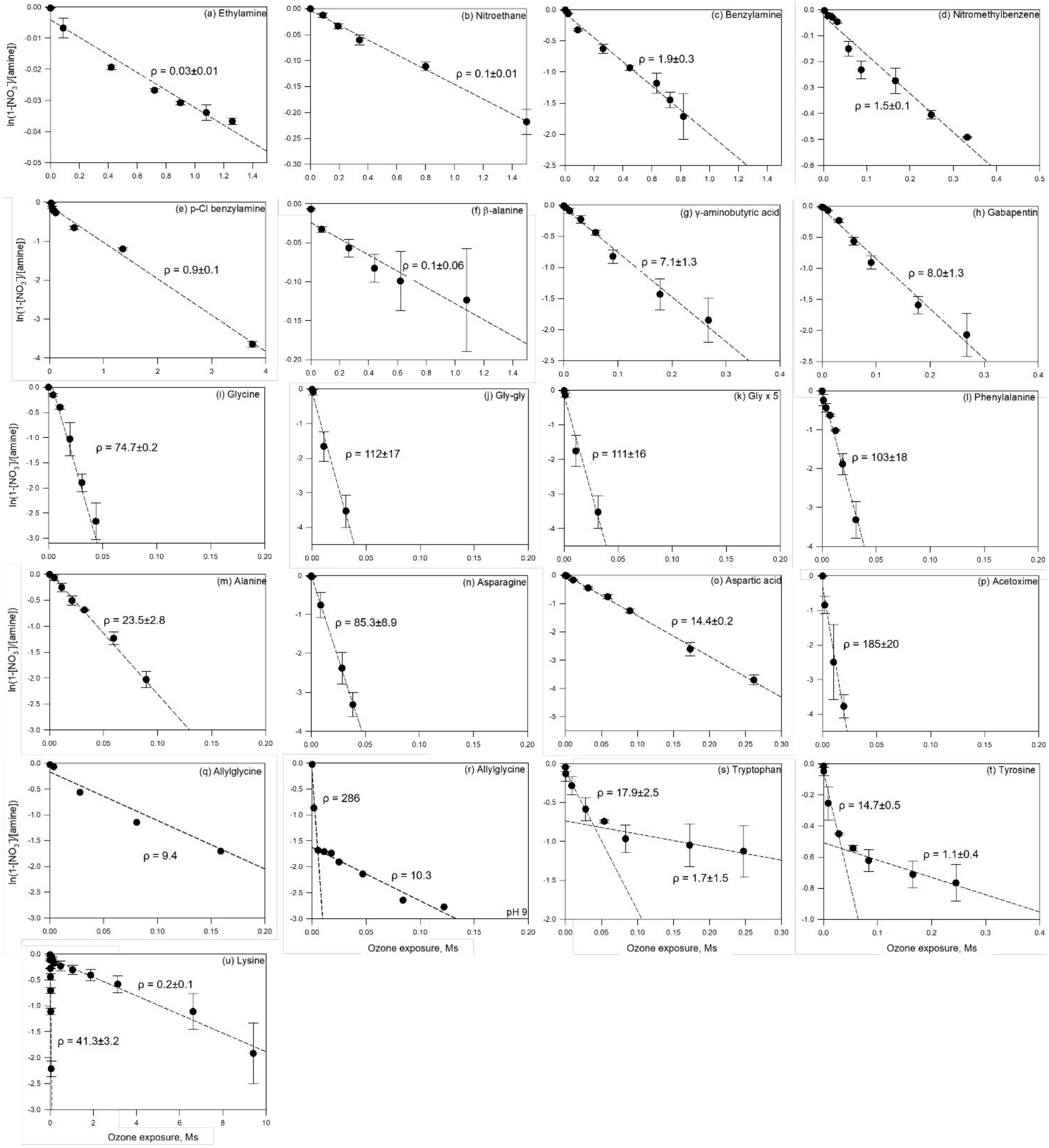

Figure S14. Continuous ozonation of model *N*-compounds. Determination of nitrate formation rate constants (k_{NO3-}, slopes (ρ) of plots of ln(1-[NO_3^-]/[model-*N*]$_0$) as a function of the ozone exposure) at pH 7 (5 mM phosphate). Dashed lines are linear regressions of data points. Error bars denote ranges of duplicate experiments with the symbols being the mean values. Experimental conditions: [model-*N*]$_0$= 5 µM (50 µM for ethylamine, nitroethane, and β-alanine, 22 µM for lysine), ozone generator power = 8% of maximum for 20 min, followed by an increase to up to 100% for 5 hours, in presence

of 50 mM *t*-BuOH.

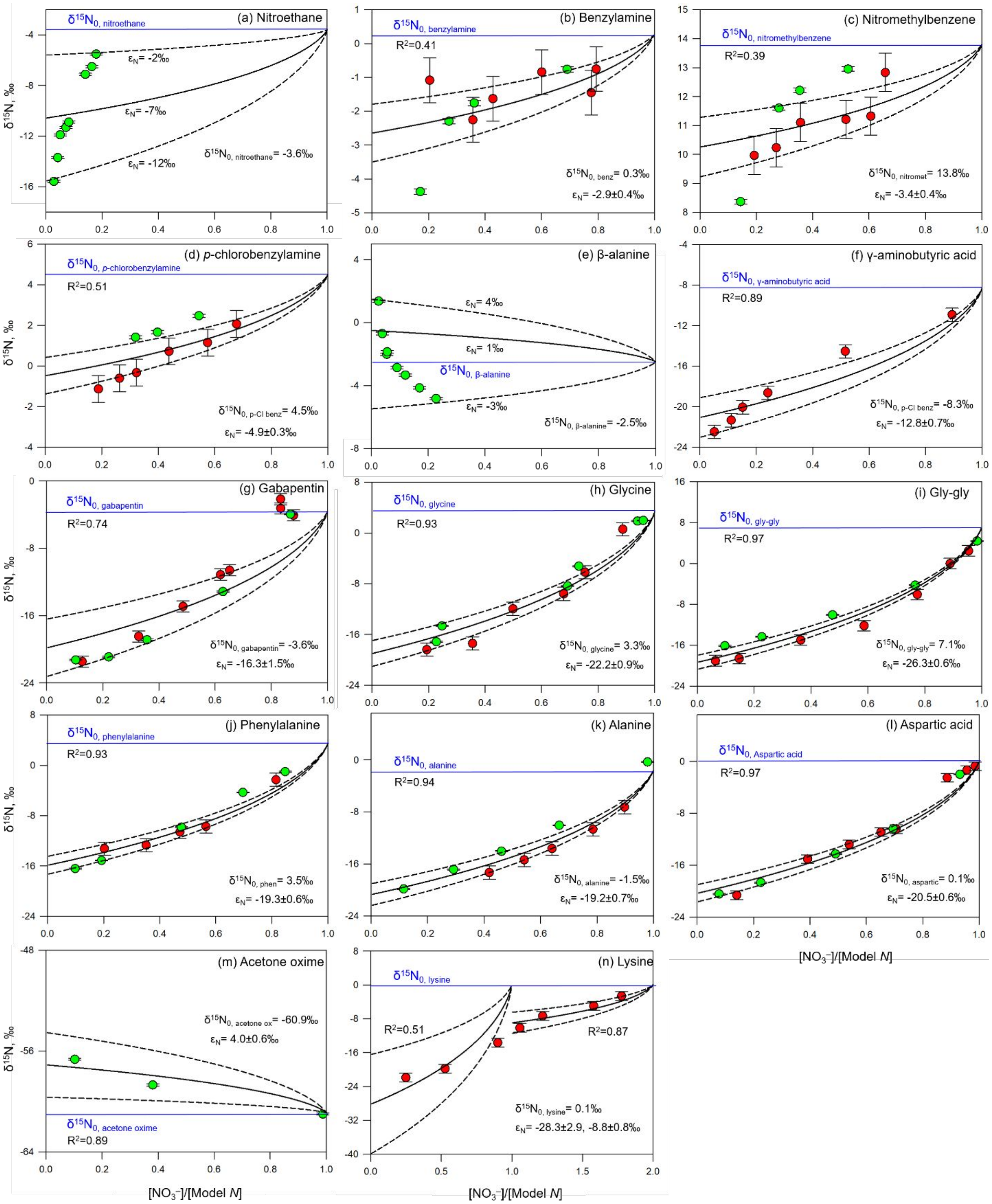

Figure S15. Continuous ozonation of selected *N*-compounds. Evolution of $\delta^{15}N$ in nitrate as a function of the conversion yield. Red and green symbols denote $\delta^{15}N$ of nitrate measured by $TiCl_3$–based conversion followed by detection with optical spectroscopy (red circles) or microbial conversion

followed by detection with GC/IR-MS (green circles), respectively. Solid lines denote ^{15}N enrichment factors (ε_N), and dashed lines denote the 95% confidence levels of the standard errors of ε_N. The error bars denote measurement errors for $\delta^{15}N$ of each instrument (± 0.4 ‰ for GC/IR-MS and ± 1.1 ‰ for laser spectroscopy). There was a slight discrepancy (< ±2 ‰) in the measured $\delta^{15}N$ of nitrate between the two different SIA methodologies. This may be due to separate experiments with different samples, the potential formation of nitrite from the hydrolysis of partially oxidized nitrogenous compounds (e.g., nitroalkane) during sample transport or deviations in $\delta^{17}O$-NO_3^- from mass-dependence affecting $\delta^{15}N$-NO_3^- analysis by IRMS. Some $\delta^{15}N$ of nitrate samples were measured using either GC/IR-MS or laser spectroscopy, but not both, due to the limitation in sample preparation or poor measurement results. Experimental conditions: [model-*N*] = 50–250 µM, ozone generator power = 8 % of maximum for 20 min, followed by an increase to up to 100% for 5 hours, in presence of 50 mM *t*-BuOH at pH 7 (5 mM phosphate).

Text S8. δ^{18}O in nitrate samples during ozonation of *N*-containing model compounds

δ^{18}O of nitrate formed during ozonation of organic nitrogen compounds mainly originates from ozone and water. In a continuous ozonation system, fresh ozone is continually supplied. Consequently, the conversion yield of dissolved ozone to nitrate cannot be assessed in a continuous ozonation system, limiting the applicability of δ^{18}O in nitrate formed from ozonation of model *N*-compounds. Figure S16 shows a plot of the δ^{15}N/δ^{18}O of nitrate samples for all selected model *N*-compounds. The δ^{18}O of nitrate in continuous ozonation of model *N*-compounds generally spanned from 40 to 60 ‰ except for compounds which already have oxygen bonded to nitrogen (e.g., nitroalkanes). All the model *N*-compounds (including the oxime) showed normal isotopic fractionation for oxygen in the formed nitrate.

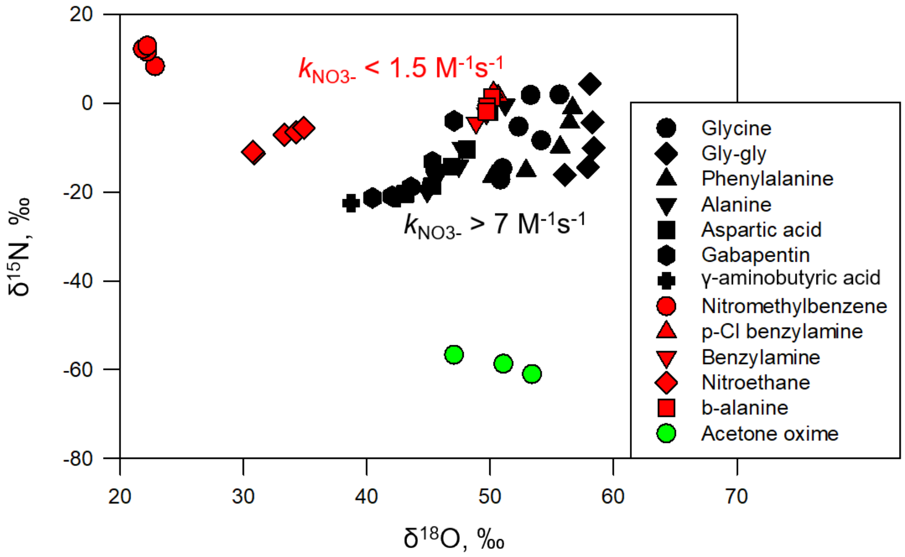

Figure S16. Plot of δ^{15}N as a function of δ^{18}O in nitrate formed during ozonation of model *N*-compounds. Red and black symbols denote aliphatic (or aryl-type) primary amines and nitroalkane (k_{NO3-}< 2 M^{-1}s^{-1}, ε_N > -5 ‰) and amino acids and peptidic amino groups (or γ-aminobutyric acid and gabapentin) (k_{NO3-}< 112 M^{-1}s^{-1}, ε_N > -28 ‰), respectively. Green symbols denote acetaldoxime with inverse nitrogen fractionation (k_{NO3-} ~185 M^{-1}s^{-1}, ε_N > 4 ‰). Experimental conditions: [model-*N*]= 50–250 µM, ozone generator power = 8 % of maximum for 20 min, followed by an increase to up to 100% for 5 hours, in presence of 50 mM *t*-BuOH at pH 7 (5 mM phosphate).

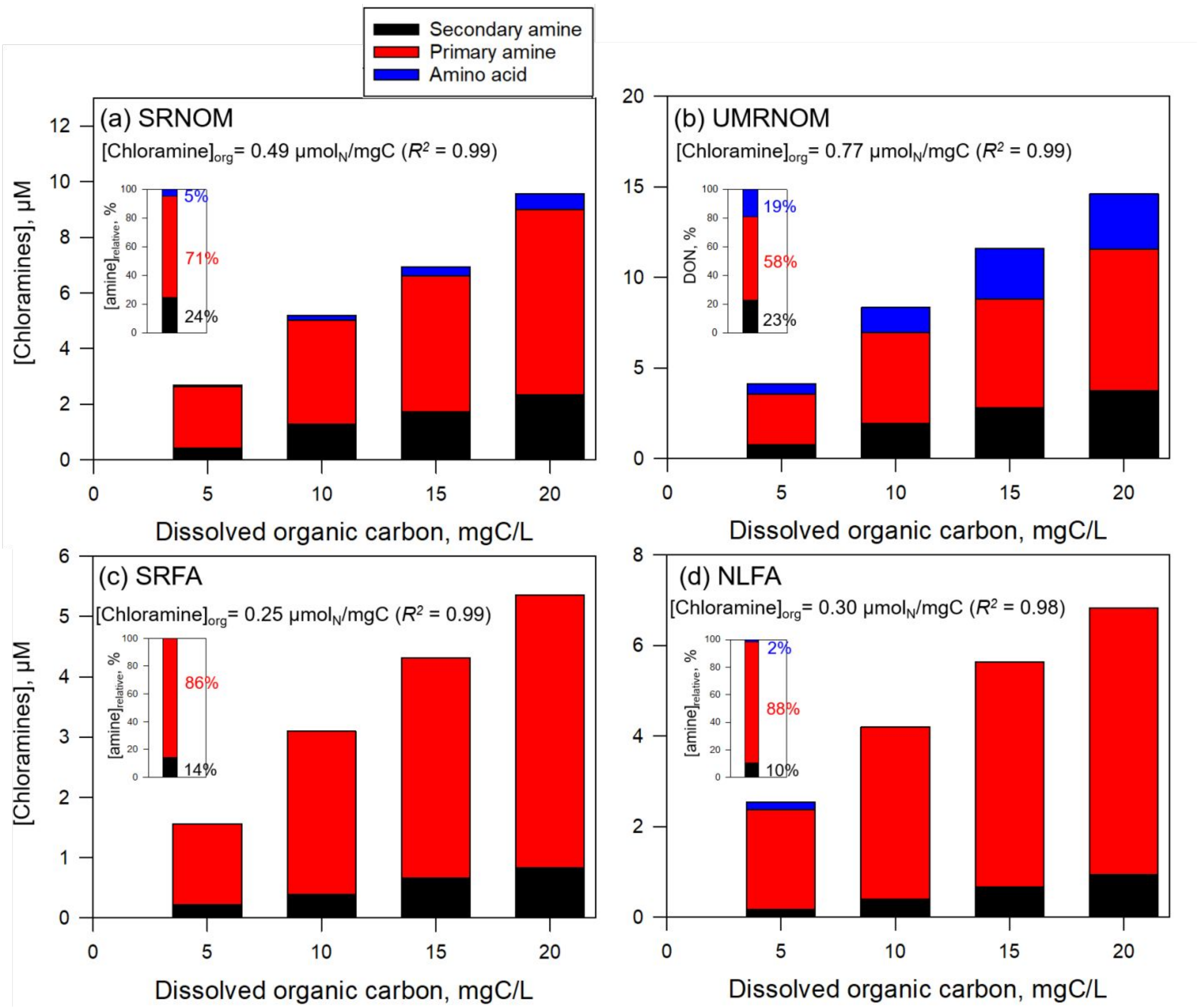

Figure S17. Chloramine formation assay: Screening of organic amine moieties in (a) SRNOM, (b) UMRNOM, (c) SRFA, and (d) NLFA for DOC concentrations in the range of 5-20 mgC/L. The inset bar chart represents the average fraction of each organic amino moiety in total chloramine for the DOC concentration range of 5-20 mgC/L. Note that the interference of ammonium was corrected from the concentrations of amino acids and primary amines (see Text S2). Experimental conditions: [FAC]= 80 μM for 2 min and 30 min, followed by addition of [resorcinol] = 10 μM for 1 min, followed by addition of [DPD] = 2 mM with [KI]= 0, 60 μM and 40 mM in sequence to measure [FAC], [*N*-Cl], and [*N*-Cl_2], respectively, at pH 6 (10 mM phosphate).

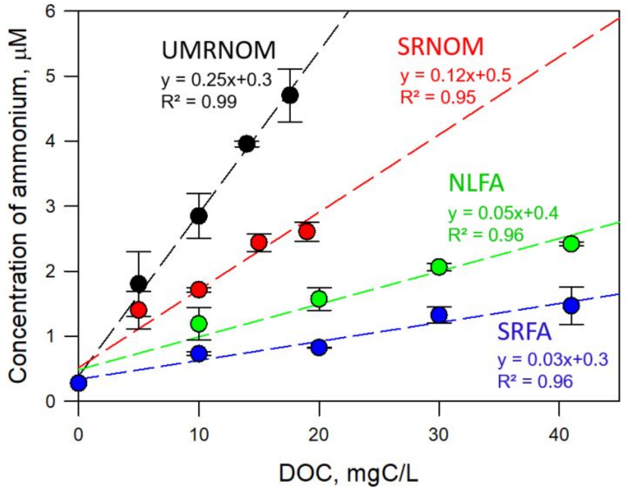

Figure S18. Determination of ammonium concentrations in NOM isolates for varying DOC concentrations (0 – 41 mgC/L). The concentration of ammonium was determined by a colorimetric method based on indophenol blue formation via the reaction with hypochlorite and phenol (Berthelot's reaction).[23] Error bars denote range of analytical duplicates with the symbols being the mean value.

Table S5. Composition (% w/w) of nitrogen and carbon in NOM isolates, and estimated DON content (International Humic Substances Society, IHSS).[13]

NOM isolates	N content (% w/w) [a]	C content (% w/w) [a]	DON content (μmolN/mgC) [b]
UMRNOM	2.4	49.9	3.4
SRNOM	1.3	50.7	1.8
SRFA	0.7	53.0	0.9
NLFA	0.7	52.3	0.9

[a] N and C are the elemental composition in % (w/w) of a dry, ash-free sample, [b] estimated from the elemental nitrogen:carbon ratio (N/C)

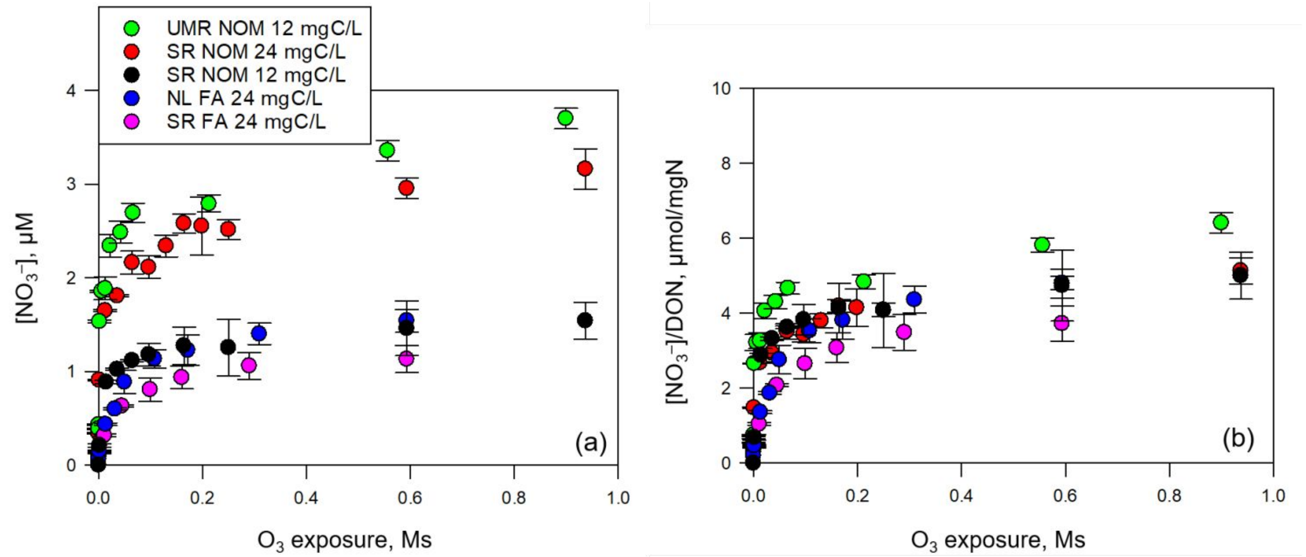

Figure S19. Evolution of (a) nitrate and (b) normalized nitrate by the DON concentration (mgN/L) of NOM isolates as a function of the ozone exposure during continuous ozonation of four natural organic matter isolates (SRNOM, UMRNOM, SRFA, NLFA). DON concentration was calculated by multiplying DOC by DON content of the NOM isolates (Table S5). Error bars denote ranges of analytical duplicates. Experimental conditions: ozone generator power = 8 % of maximum, 50 mM *t*-BuOH at pH 7 (5 mM phosphate).

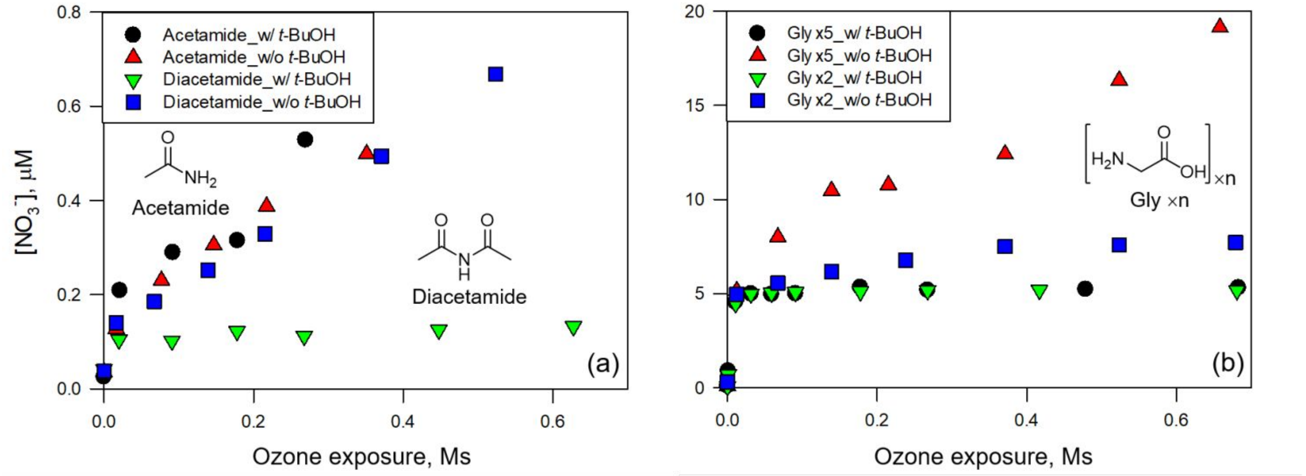

Figure S20. Evolution of nitrate as a function of the ozone exposure during continuous ozonation of (a) amide model compounds and (b) glycine-derived peptidic compounds in absence or presence of *t*-BuOH. Experimental conditions: [acetamide] = [diacetamide] = 10 μM, [gly×2] = [gly×5]= 5 μM in presence of 50 mM *t*-BuOH at pH 7 (5 mM phosphate).

Table S6. Fraction of each reactive amine moiety in SRNOM and UMRNOM (% for SRNOM / % for UMRNOM), quantified by three complementary approaches in percentage of the estimated DON. The DON contents of the two NOM isolates are 1.8 µmolN/mgC and 3.4 µmolN/mgC, respectively (Table S5). Errors denote standard deviation of different measurements or analytical duplicates. For more details on the calculations see main text (section 3.3.4).

<table>
<tr><th>Reactive amine moieties</th><th>Chloramine formation assay, %</th><th>Nitrate formation assay, %</th><th>SIA of nitrate, %</th></tr>
<tr><td>Secondary amines</td><td>6±1.0 [a] / 5±0.5 [b]</td><td rowspan="2">3.0±0.4 / 3.0±0.3</td><td rowspan="3">7±0.4 / 7±0.4</td></tr>
<tr><td>Aliphatic primary amines</td><td rowspan="3">20±2.9 / 14±2.3</td></tr>
<tr><td>Aryl-type primary amines</td><td>4.0±0.4 / 4.0±0.5</td></tr>
<tr><td>Peptidic amino groups</td><td rowspan="2">3.0±0.3 / 4.0±0.4</td><td rowspan="2">3±0.3 / 4.0±0.4</td></tr>
<tr><td>Amino acids</td><td>1±0.4 / 4±0.8</td></tr>
<tr><td>Sum</td><td>27 / 23</td><td>10 / 11</td><td>10 / 11</td></tr>
</table>

[a] Fractions in SRNOM, [b] fractions in UMRNOM

References

1. Mohn, J.; Biasi, C.; Bodé, S.; Boeckx, P.; Brewer, P. J.; Eggleston, S.; Geilmann, H.; Guillevic, M.; Kaiser, J.; Kantnerová, K., Isotopically characterised N2O reference materials for use as community standards. *Rapid Communications in Mass Spectrometry* **2022,** *36* (13), e9296.
2. Deborde, M.; von Gunten, U., Reactions of chlorine with inorganic and organic compounds during water treatment—kinetics and mechanisms: a critical review. *Water research* **2008,** *42* (1-2), 13-51.
3. Ra, J.; Lee, Y., Efficient degradation of ethanolamine by UV/chlorine process via organic chloramine photolysis: Kinetics, products, and implications for ethanolamine wastewater treatment. *Chemical Engineering Journal* **2021,** *412*, 128631.
4. Mitch, W. A.; Schreiber, I. M., Degradation of tertiary alkylamines during chlorination/chloramination: Implications for formation of aldehydes, nitriles, halonitroalkanes, and nitrosamines. *Environmental science technology* **2008,** *42* (13), 4811-4817.
5. Ra, J.; Yoom, H.; Son, H.; Hwang, T.-M.; Lee, Y., Transformation of an amine moiety of atenolol during water treatment with chlorine/UV: reaction kinetics, products, and mechanisms.

Environmental Science Technology **2019,** *53* (13), 7653-7662.
6. How, Z. T.; Kristiana, I.; Busetti, F.; Linge, K. L.; Joll, C. A., Organic chloramines in chlorine-based disinfected water systems: A critical review. *J. Environ. Sci.* **2017,** *58*, 2-18.
7. Joo, S. H.; Mitch, W. A., Nitrile, aldehyde, and halonitroalkane formation during chlorination/chloramination of primary amines. *Environ. Sci. Technol.* **2007,** *41* (4), 1288-1296.
8. Lipps WC, B. T., Braun-Howland E, editors, *4500-cl chlorine (residual) In: Standard Methods For the Examination of Water and Wastewater*. Washington DC: APHA Press.
9. Cimetiere, N.; Dossier-Berne, F.; De Laat, J., Monochloramination of resorcinol: mechanism and kinetic modeling. *Environmental science technology* **2009,** *43* (24), 9380-9385.
10. Rebenne, L. M.; Gonzalez, A. C.; Olson, T. M., Aqueous chlorination kinetics and mechanism of substituted dihydroxybenzenes. *Environmental Science Technology* **1996,** *30* (7), 2235-2242.
11. Wei, I., Dynamics of breakpoint chlorination. **1974**.
12. Granger, J.; Sigman, D. M., Removal of nitrite with sulfamic acid for nitrate N and O isotope analysis with the denitrifier method. *Rapid Communications in Mass Spectrometry: An International Journal Devoted to the Rapid Dissemination of Up‐to‐the‐Minute Research in Mass Spectrometry* **2009,** *23* (23), 3753-3762.
13. International Humic Substances Society. Elemental Compositions and Stable Isotopic Ratios of IHSS Samples. https://humic-substances.org/elemental-compositions-and-stable-isotopic-ratios-of-ihss-samples/ (accessed 2023-06-17).
14. Houska, J.; Salhi, E.; Walpen, N.; von Gunten, U., Oxidant-reactive carbonous moieties in dissolved organic matter: Selective quantification by oxidative titration using chlorine dioxide and ozone. *Water Research* **2021,** *207*, 117790.
15. UC Davis Stable Isotope Facility. Nitrate (NO3) in Water. https://stableisotopefacility.ucdavis.edu/nitrate-no3-water (accessed 2022-02-15).
16. University of Calgary Isotope Science Lab. δ15N and δ18O isotopic analysis by "bacterial denitrifier" method. https://www.ucalgary.ca/sites/default/files/teams/261/01_denitrifier-delta_TON.pdf (accessed 2023-03-12).
17. Altabet, M. A.; Wassenaar, L. I.; Douence, C.; Roy, R., A Ti (III) reduction method for one-step conversion of seawater and freshwater nitrate into N2O for stable isotopic analysis of 15N/14N, 18O/16O and 17O/16O. *Rapid Communications in Mass Spectrometry* **2019,** *33* (15), 1227-1239.
18. Huang, K.; Eschenbach, W.; Wei, J.; Hausherr, D.; Frey, C.; Kupferschmid, A.; Dyckmans, J.; Joss, A.; Lehmann, M. F.; Mohn, J., Simultaneous 15N Online Analysis in NH4+, NO2–, NO3–, and N2O to Trace N2O Production Pathways in Nitrogen-Polluted Aqueous Environments. *ACS EST Water* **2023,** *3* (11), 3485-3495.
19. Ibraim, E.; Harris, E.; Eyer, S.; Tuzson, B.; Emmenegger, L.; Six, J.; Mohn, J., Development of a field-deployable method for simultaneous, real-time measurements of the four most abundant N2O isotopocules. *Isotopes in environmental health studies* **2018,** *54* (1), 1-15.
20. Harris, S. J.; Liisberg, J.; Xia, L.; Wei, J.; Zeyer, K.; Yu, L.; Barthel, M.; Wolf, B.; Kelly, B. F.; Cendón, D. I., N 2 O isotopocule measurements using laser spectroscopy: analyzer characterization and intercomparison. *Atmospheric Measurement Techniques* **2020,** *13* (5), 2797-2831.
21. Mohn, J.; Wolf, B.; Toyoda, S.; Lin, C. T.; Liang, M. C.; Brüggemann, N.; Wissel, H.; Steiker, A. E.; Dyckmans, J.; Szwec, L., Interlaboratory assessment of nitrous oxide isotopomer analysis by isotope ratio mass spectrometry and laser spectroscopy: current status and perspectives. *Rapid communications in mass spectrometry* **2014,** *28* (18), 1995-2007.
22. Bader, H.; Sturzenegger, V.; Hoigné, J., Photometric method for the determination of low

concentrations of hydrogen peroxide by the peroxidase catalyzed oxidation of N, N-diethyl-p-phenylenediamine (DPD). *Water Research* **1988,** *22* (9), 1109-1115.
23. Patton, C. J.; Crouch, S., Spectrophotometric and kinetics investigation of the Berthelot reaction for the determination of ammonia. *Analytical chemistry* **1977,** *49* (3), 464-469.
